# Supplementary material for: Magainin 2 and PGLa in bacterial membrane mimics IV: Membrane curvature and partitioning
Source: Biophys J. 2022 Oct 18;121(23):4689–701. doi: 10.1016/j.bpj.2022.10.018 (PMC9748257; doi:10.1016/j.bpj.2022.10.018)
Supplement: Document S1. Tables S1–S3 and Figures S1–S16 [file mmc1.pdf]

Biophysical Journal, Volume *121*

Supplemental information

Magainin 2 and PGLa in bacterial membrane mimics IV: Membrane curvature and partitioning

Enrico F. Semeraro, Peter Pajtinka, Lisa Marx, Ivo Kabelka, Regina Leber, Karl Lohner, Robert Vácha, and Georg Pabst

SUPPLEMENTARY MATERIAL

Magainin 2 and PGLa in Bacterial Membrane Mimics IV: Membrane Curvature and Partitioning

E.F. Semeraro, P. Patik, L. Mark, I. Kableka, R. Leber, K. Lohner, R. Vácha, and G. Pabst

Table S1: List of simulated egg-box bilayers. The overall simulation length, number of peptide copies per leaflet, and height amplitude, Amp, is listed.

Peptide	Amp [nm]	#Replicas	Length [μs]	#Peptides per leaflet
MG2a	1.0	1	10.5	2
MG2a	1.0	2	6.75	2
MG2a	2.5	1	10.5	2
MG2a	2.5	2	4.5	2
MG2a	4.0	1	10.5	2
MG2a	4.0	2	4.5	2
L18W-PGLa	1.0	1	10.5	2
L18W-PGLa	1.0	2	6.75	2
L18W-PGLa	2.5	1	10.5	2
L18W-PGLa	2.5	2	4.5	2
L18W-PGLa	4.0	1	10.5	2
L18W-PGLa	4.0	2	4.5	2
MG2a:MG2a	2.5	1	10.5	4 (2 dimers)
L18W-PGLa:L18W-PGLa	2.5	1	10.5	4 (2 dimers)
L18W-PGLa:MG2a	2.5	1	10.5	4 (2 dimers)
L18W-PGLa:MG2a	2.5	2	5.25	4 (2 dimers)

Table S2: Peptide-induced changes of mean torque, $\Delta\tau$, and spontaneous monolayer curvature, Δc_0^m for peptides with imposed fully or partial α-helical secondary structure. Reported values are averaged over both symmetric lipid leaflets.

Peptides	$\Delta\tau$ [10^{-12} J m^{-1}]	Δc_0^m [nm^{-1}]
MG2a[a,c]	1.88 ± 0.15	0.029 ± 0.003
MG2a[a,d]	1.7 ± 0.3	0.027 ± 0.004
PGLa[a,c]	0.98 ± 0.18	0.015 ± 0.003
PGLa[a,e]	0.92 ± 0.11	0.014 ± 0.002
2x MG2a[b,c]	3.7 ± 0.6	0.057 ± 0.009
2x MG2a[b,d]	3.1 ± 0.5	0.049 ± 0.007
2x PGLa[b,c]	2.3 ± 0.9	0.036 ± 0.014
2x PGLa[b,e]	2.58 ± 0.16	0.040 ± 0.003
L18W-PGLa:MG2a[b,c,c]	2.75 ± 0.19	0.043 ± 0.004
L18W-PGLa:MG2a[b,e,d]	2.77 ± 0.14	0.043 ± 0.003

[a] monomer.
[b] dimer
[c] 100% helicity.
[d] 74% helicity.
[e] 76% helicity.

Table S3: Average value of the sensed mean membrane curvature, H, by studied peptides with different helical content. Standard errors were calculated from sampled curvatures by peptides on different leaflets and different systems.

Peptides	H [nm^{-1}]
MG2a (monomer)[a]	0.0848 ± 0.0006
MG2a (monomer)[b]	0.0821 ± 0.0013
PGLa (monomer)[a]	0.065 ± 0.002
PGLa (monomer)[c]	0.075 ± 0.002
2x MG2a (dimer)[a,d]	0.1033 ± 0.0003
2x MG2a (dimer)[b]	0.1003 ± 0.0007
2x PGLa (dimer)[a]	0.0859 ± 0.0004
2x PGLa (dimer)[c]	0.0933 ± 0.0010
L18W-PGLa:MG2a (dimer)[a,d]	0.1017 ± 0.0003
L18W-PGLa:MG2a (dimer) [c,b,d]	0.0990 ± 0.0006

[a] 100% helicity.
[b] 74% helicity.
[c] 76% helicity.
[d] One of the dimers dissociated during the simulation run and was therefore discarded from the analysis.

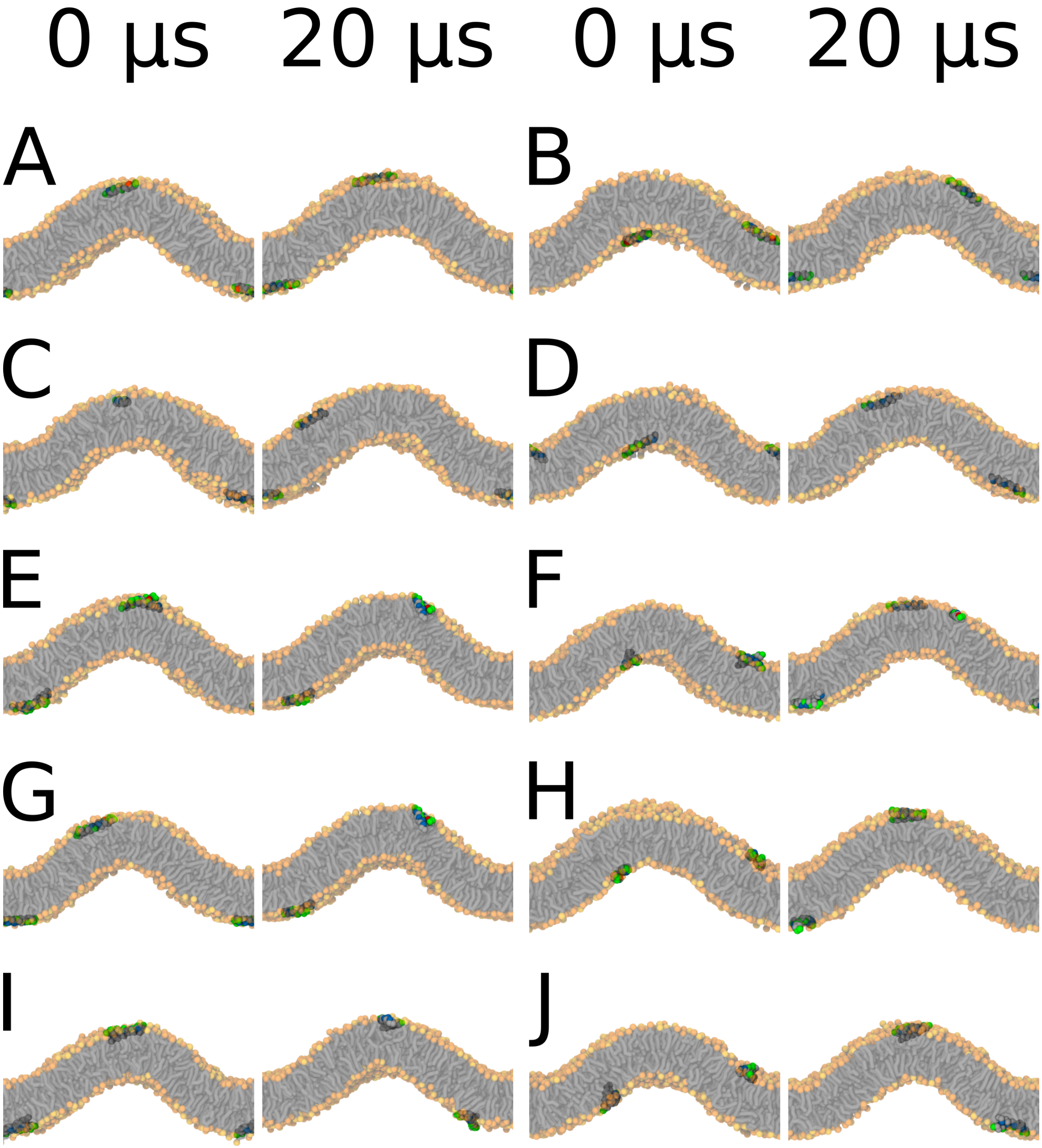

Figure S1: Initial and final simulation snapshots of peptide localization on a curved PE:PG (3:1 mol/mol) membrane. (*A*–*B*) two different initial configurations of systems comprising monomeric MG2a on each membrane leaflet (*C*–*D*) two different initial configurations of monomeric L18W-PGLa, one on each membrane leaflet. (*E*–*F*) two different initial configurations of systems with heterodimer MG2a:L18W-PGLa, one heterodimer on each leaflet (*G*–*H*) two different initial configurations of systems with MG2a homodimer, one homodimer on each leaflet (*I*–*J*) two different initial configurations of systems with L18W-PGLa homodimer, one homodimer on each leaflet.

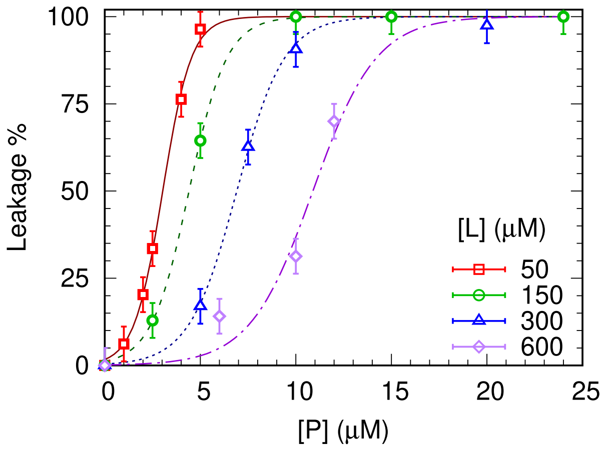

Figure S2: Leakage percentage of the dye-quencher pair ANTS/DPX from POPE/POPG 3:1 lipid vesicles as a function L18W-PGLa concentration, $[P]$, at different total lipid concentrations $[L]$. Data were fitted with a sigmoidal function by fixing a final plateau at 100%.

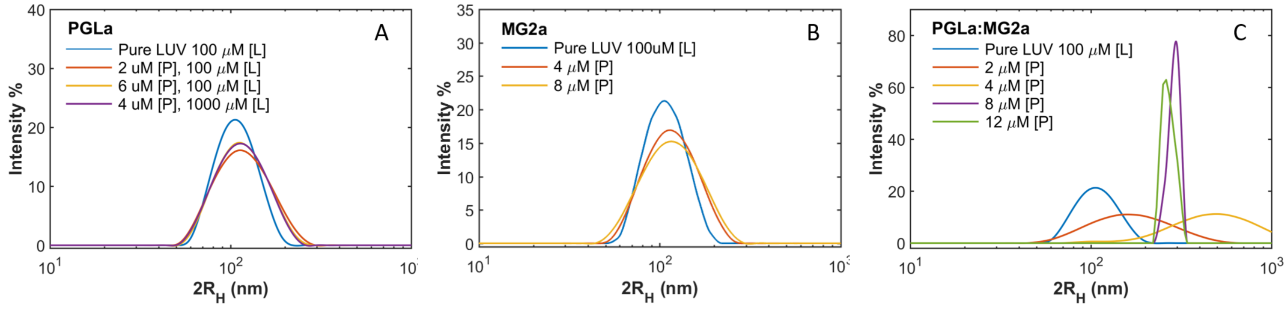

Figure S3: Representative size distribution functions (intensity-weighted) of POPE/POPG 3:1 membrane mimics after incubation with peptides for one hour at 37 °C. **A:** LUVs ($[L]$=100 or 1000 μM) after incubation with PGLa ($[P]$=2-6 μM). **B:** ($[P]$=4-8 μM) MG2a mixed with LUVs ($[L]$=100 μM). **C:** ($[P]$=2-12 μM) PGLa:MG2a mixed with LUVs ($[L]$=100 μM).

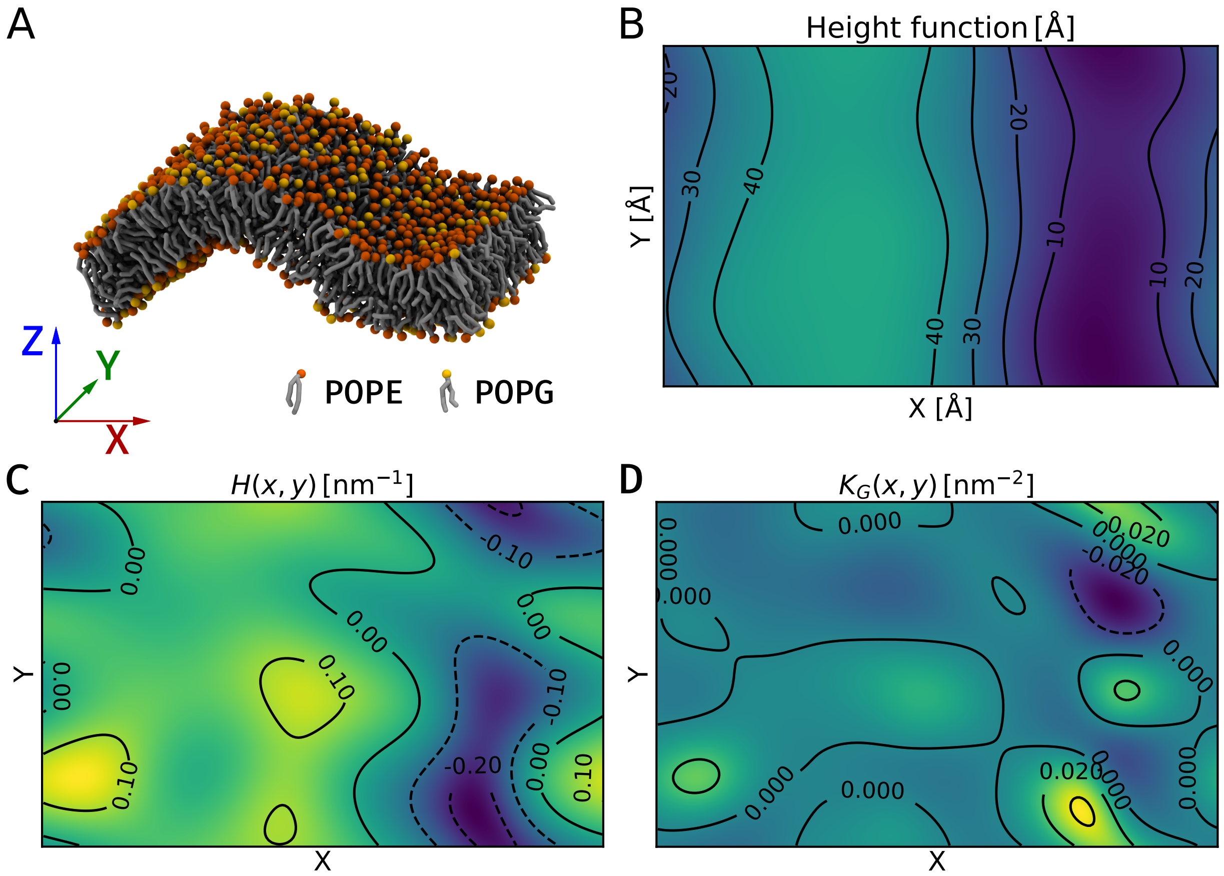

Figure S4: A snapshot of buckled bilayer system (*A*) with a corresponding map of height function (*B*), map of mean curvature (*C*), and a map of Gaussian curvature (*D*). In the snapshot, solvent, ion, and protein beads are omitted for clarity. The maps shown here correspond to the upper membrane leaflet.

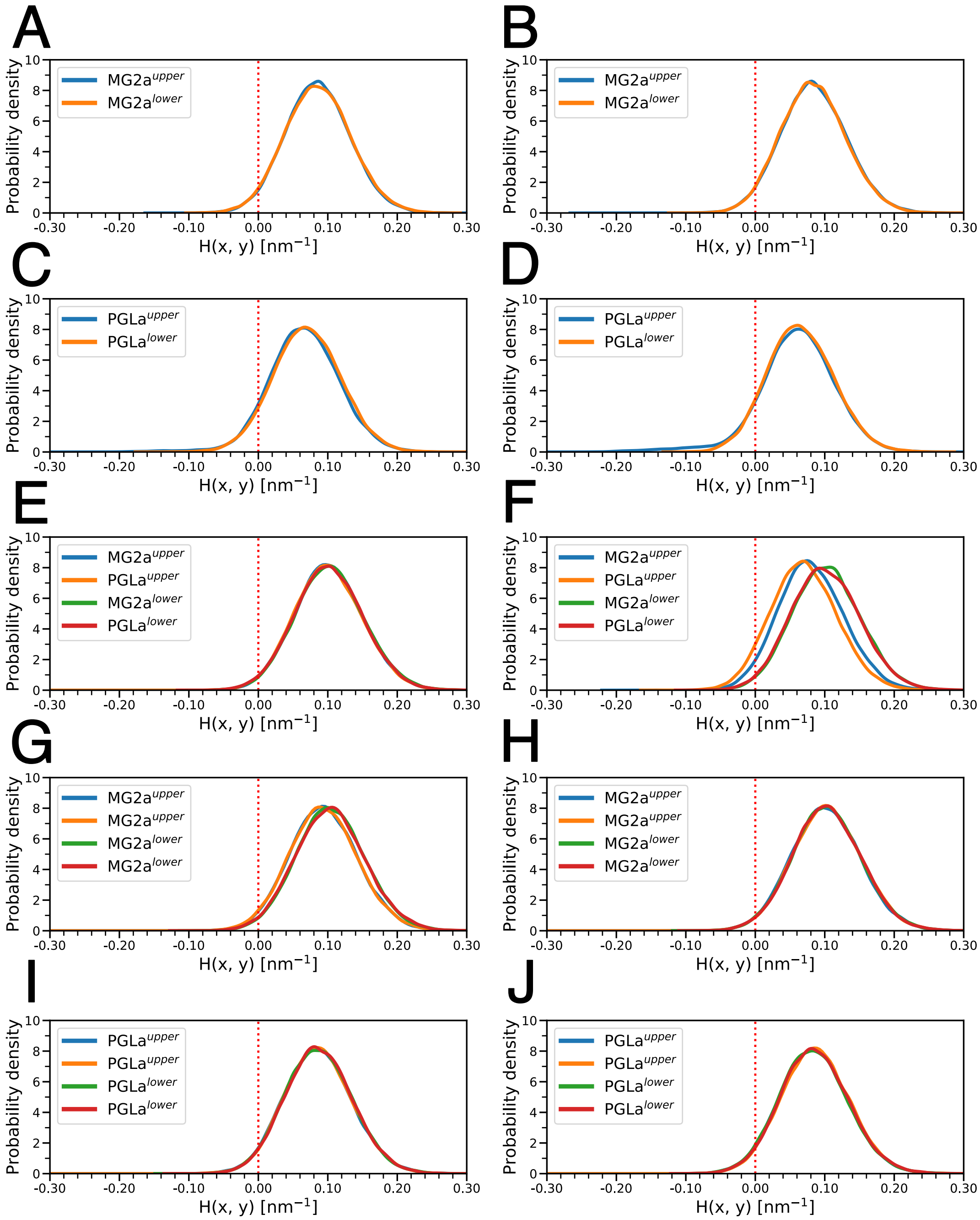

Figure S5: Kernel density estimates from the histograms of the sampled mean curvature by peptides, with fully α-helical secondary structure, on the buckled PE:PG (3:1 mol/mol) membrane. The red dotted-line highlights the zero mean curvature. (*A*–*B*) two different initial configurations of systems comprising monomeric MG2a on each membrane leaflet. (*C*–*D*) two different initial configurations of monomeric L18W-PGLa, one on each membrane leaflet. (*E*–*F*) two different initial configurations of systems with heterodimer MG2a:L18W-PGLa, one heterodimer on each leaflet. In the second system, one copy of heterodimer disassembled (after approximately 4 μs), resulting in a different curvature sampled by the MG2a and L18W-PGLa monomers. (*G*–*H*)-two different initial configurations of homodimer of MG2a. Similarly as in the case of heterodimer, one copy of dimer dissociated (after approx. 12 μs) and different mean curvatures sampled by the monomers caused slight shift in the distribution towards lower values of mean curvature. (*I*–*J*)-two initial configurations of PGLa homodimer.

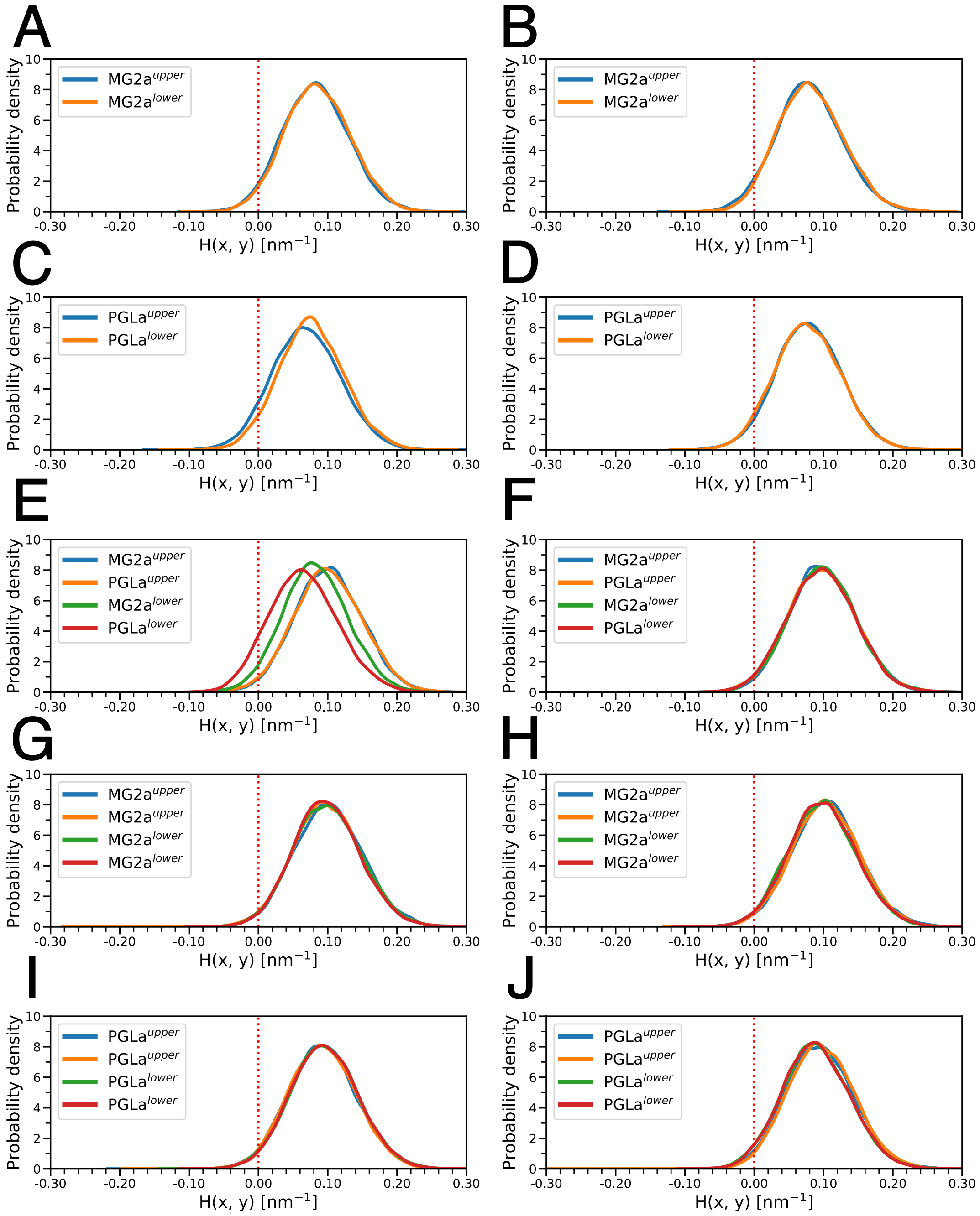

Figure S6: Kernel density estimates from the histograms of the sampled mean curvature by peptides, with partially unfolded secondary structure (for details see Methods section), on the buckled PE:PG (3:1 mol/mol) membrane. The red dotted-line highlights the zero mean curvature. (*A–B*) two different initial configurations of systems comprising monomeric MG2a on each membrane leaflet. (*C–D*) two different initial configurations of monomeric L18W-PGLa, one on each membrane leaflet. (*E–F*) two different initial configurations of systems with heterodimer MG2a:L18W-PGLa, one heterodimer on each leaflet. In the first system, one copy of heterodimer disassembled within the first microsecond, resulting in a different curvature sampled by the MG2a and L18W-PGLa monomers. (*G–H*)-two different initial configurations of homodimer of MG2a. (*I–J*)-two initial configurations of PGLa homodimer.

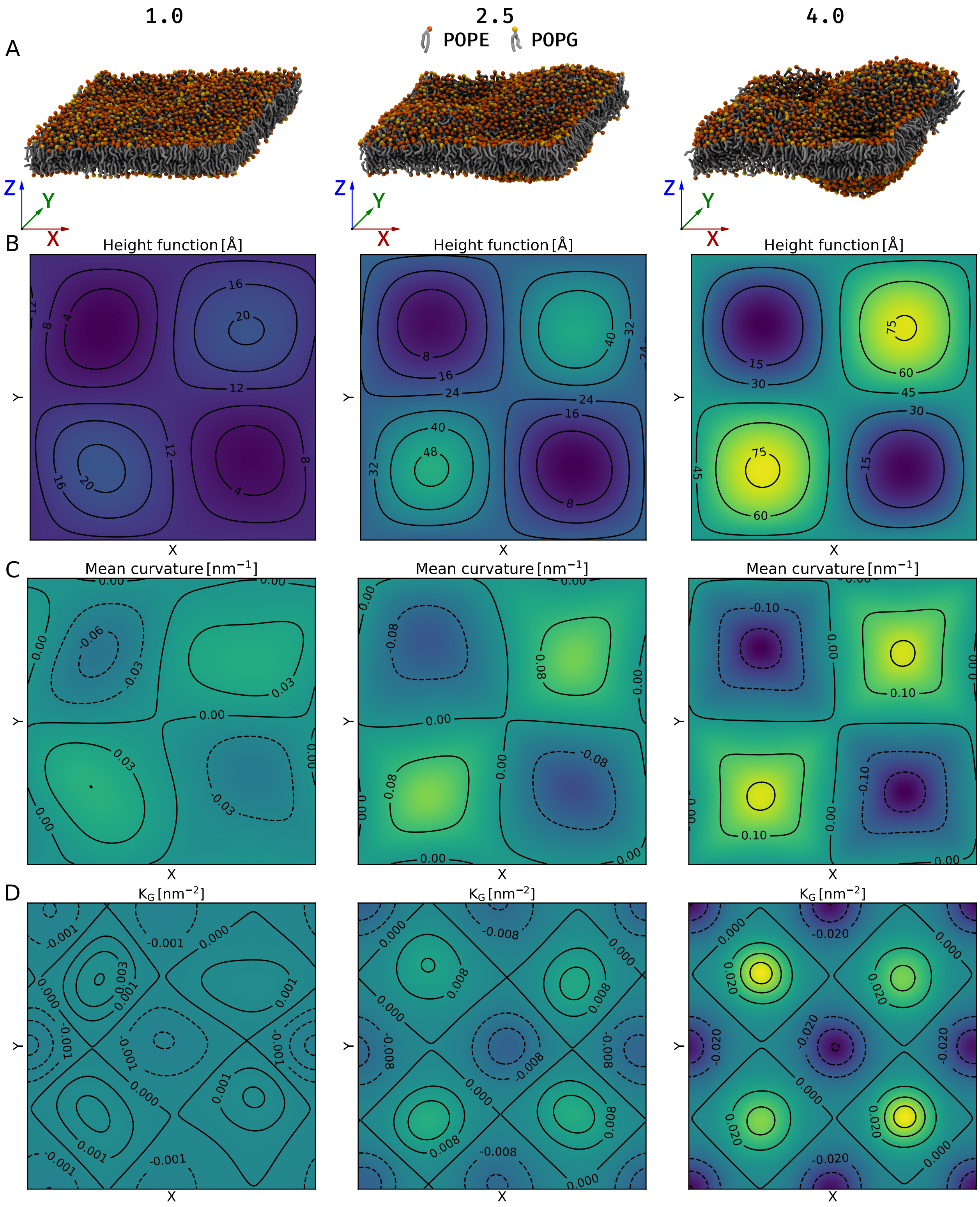

Figure S7: An illustrative summary of the egg-box bilayer systems with different height amplitudes (1.0, 2.5, and 4.0 nm) and corresponding accessible curvatures. In the top row (*A*), snapshots for systems of different amplitudes are shown. The corresponding maps of height function (*B*), mean curvature distribution (*C*), and Gaussian curvature distribution (*D*) for each of the amplitudes are shown in the appropriate column.

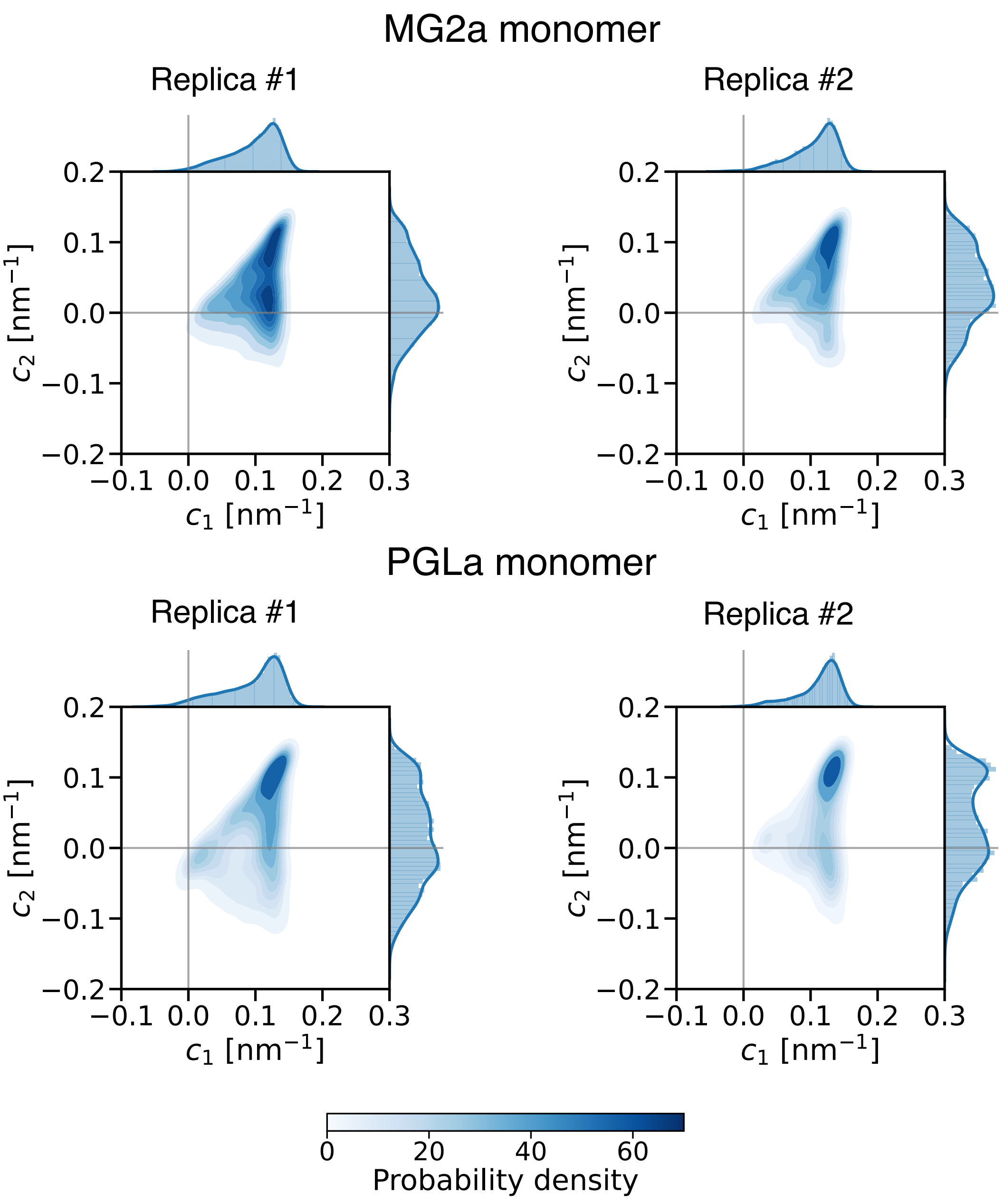

Figure S8: 2D histograms of principal curvatures c_1 and c_2 sampled by the monomeric MG2a and PGLa peptides on the egg-box shaped bilayer. Results obtained from both replicas are presented side by side. Gaussian curvature, K_G, is defined as $c_1 c_2$.

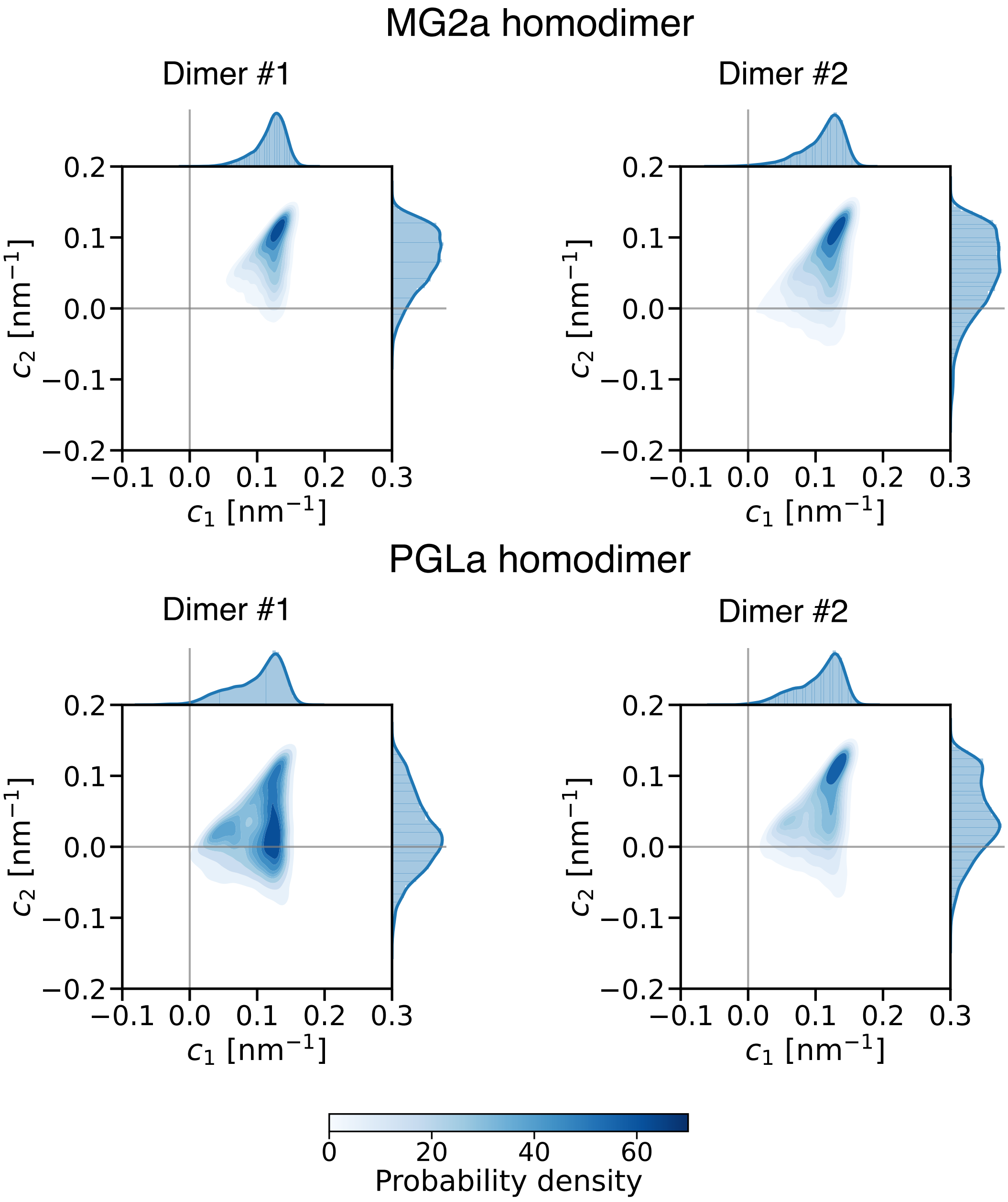

Figure S9: 2D histograms of principal curvatures c_1 and c_2 sampled on the egg-box shaped bilayer by the homodimeric MG2a and PGLa peptides, respectively. Results obtained from both dimers present on unbiased leaflet are shown side by side. Gaussian curvature, K_G, is defined as $c_1 c_2$.

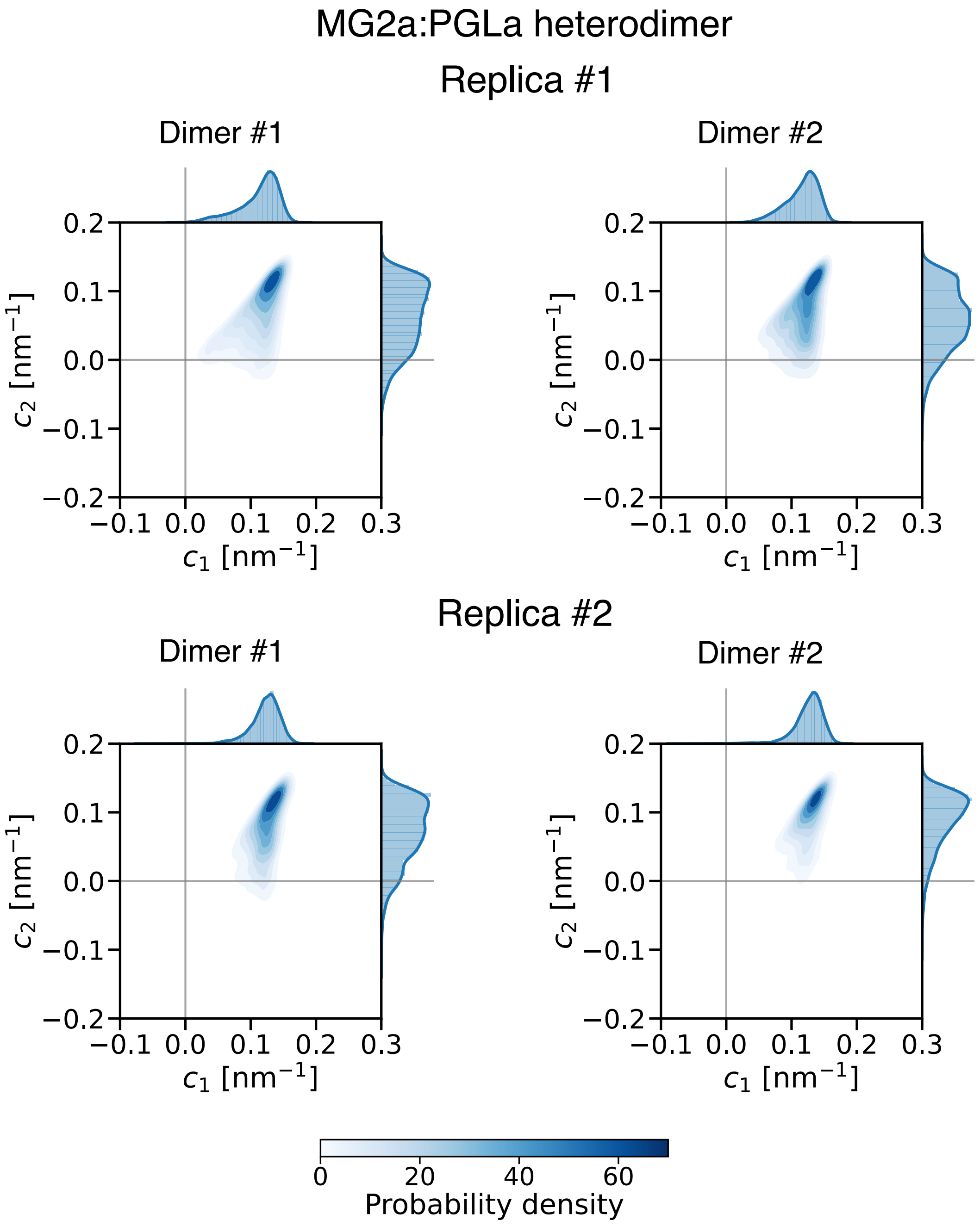

Figure S10: 2D histograms of principal curvatures c_1 and c_2 sampled on the egg-box shaped bilayer by the MG2a:PGLa heterodimer. Results obtained from both replicas and from both dimers present at unbiased membrane leaflets are shown. Gaussian curvature, K_G, is defined as c_1c_2.

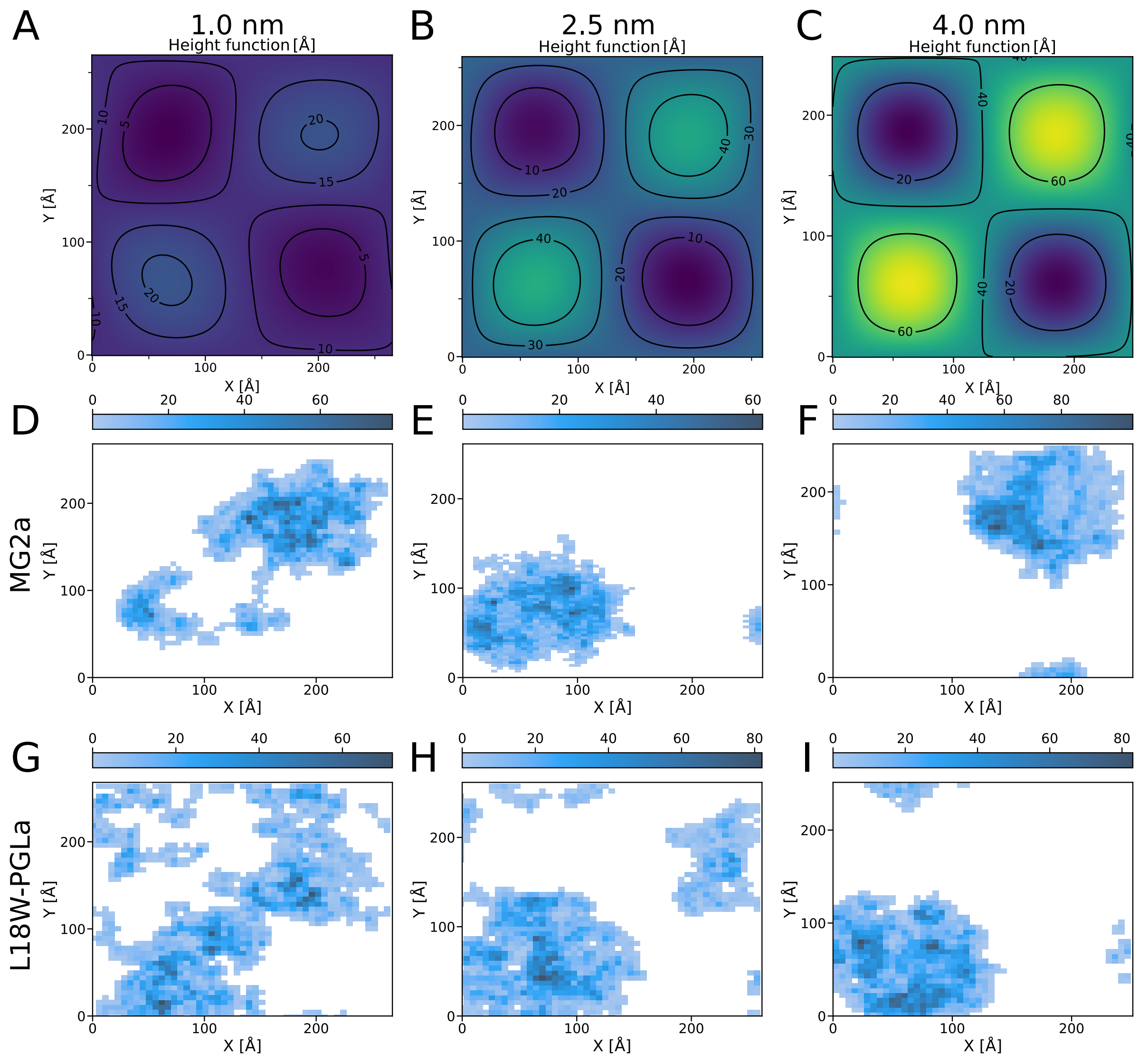

Figure S11: Comparison of the systems with egg-box shaped membranes of different amplitudes (1.0 nm, 2.5 nm, or 4.0 nm). To investigate the effect, systems containing one copy of monomeric MG2a or L18W-PGLa were used. (*A*-*C*) Height functions, representing the shape of the upper leaflet for different amplitudes are shown. 2D histograms of XY coordinates MG2a center of mass sampled during the simulation run at membrane with amplitude 1.0 nm (*D*), 2.5 nm (*E*), and 4.0 nm (*F*). 2D histogram of XY coordinates L18W-PGLa center of mass visited during the simulation run on membrane with 1.0 nm (*G*), 2.5 nm (*H*), and 4.0 nm (*I*) amplitude.

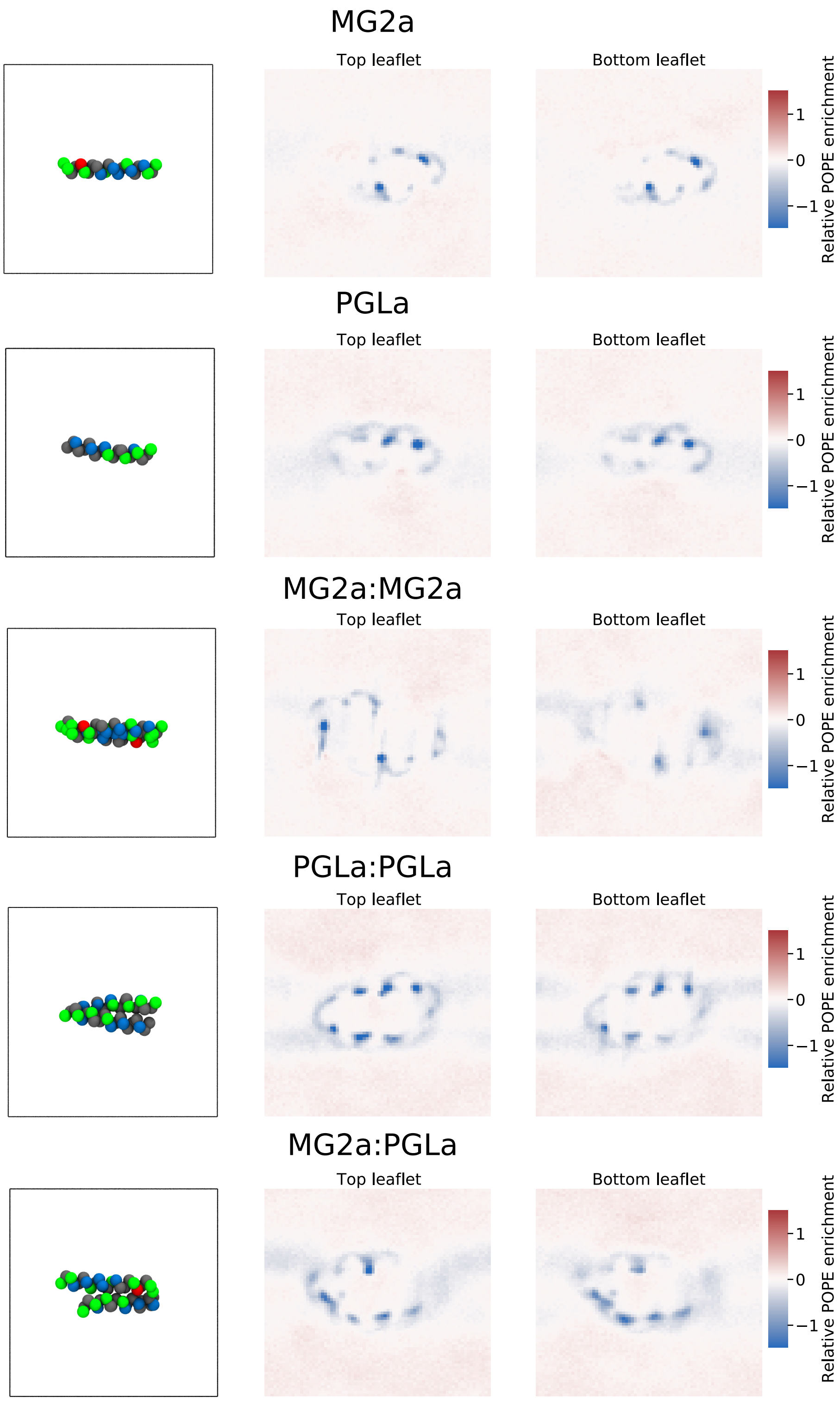

Figure S12: 2D averaged density maps of a planar POPE:POPG (3:1 mol:mol) membrane with peptides averaged over an 8μs trajectory, showing relative POPE enrichment in the proximity of the peptides. Negative POPE enrichment corresponds to the enrichment of POPG in the particular region. The systems were centered and aligned on peptides as indicated by the snapshots. The enrichment was calculated as a difference between normalized density maps of PO_4 beads from POPE and POPG lipids. Data were averaged over both membrane leaflets.

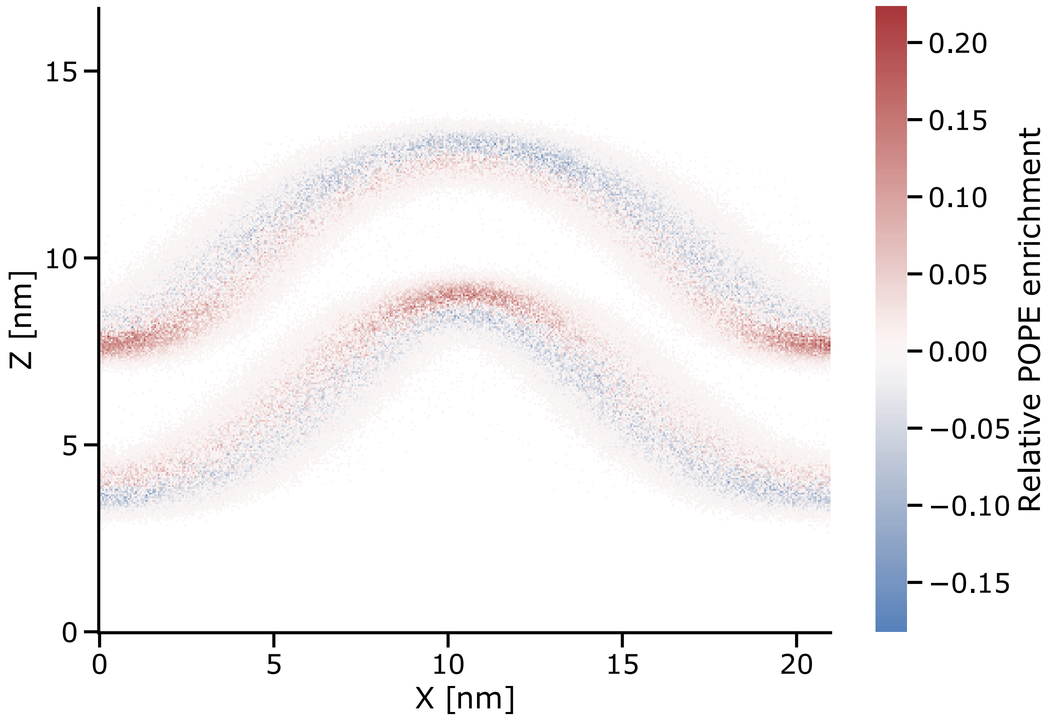

Figure S13: Density map of a membrane POPE:POPG (3:1 mol:mol) buckle without peptides averaged over Y direction, showing relative POPE enrichment within differently curved membrane regions. The enrichment was calculated as a difference between normalized density profiles of POPE and POPG lipids. Note that only densities of PO4 beads are shown.

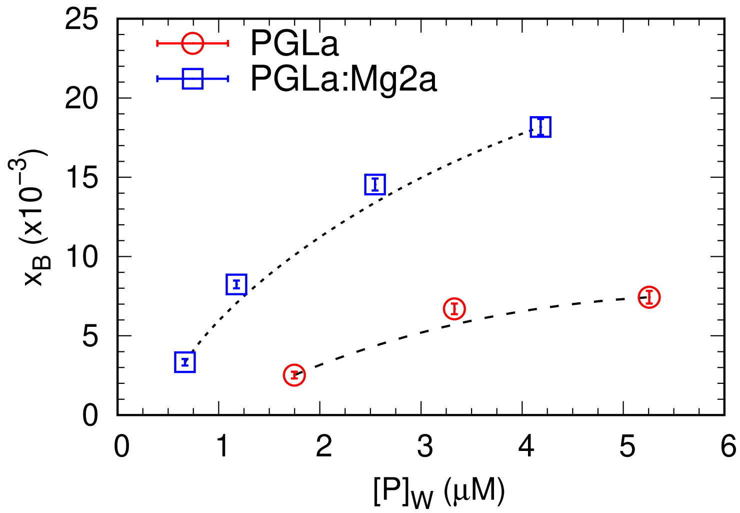

Figure S14: Number of partitioned L18W-PGLa per lipid molecule, x_B, as a function of free PGLa in bulk, $[P]_W$, for $[L]$ = 100 μM at different PGLa concentrations. Dashed lines are guides for the eyes.

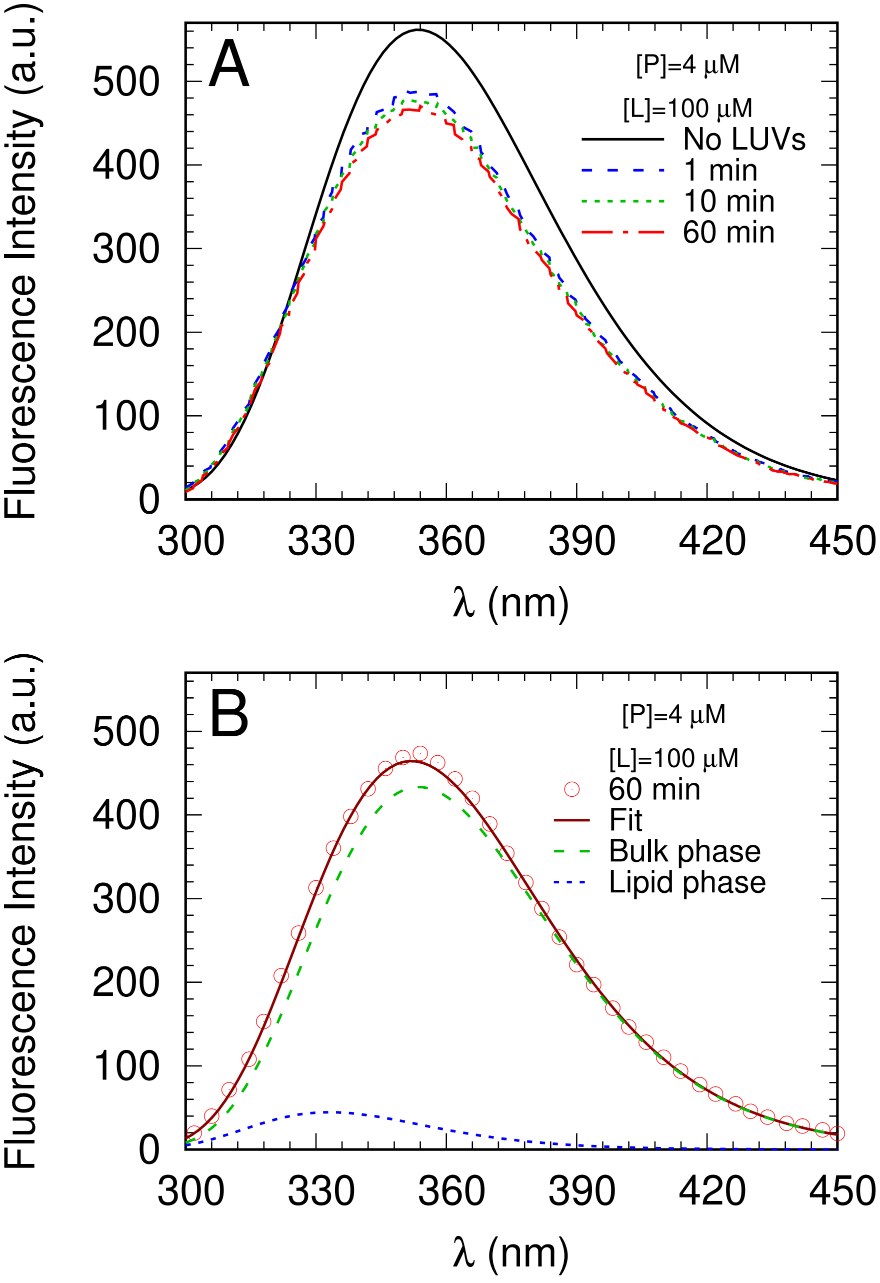

Figure S15: **A:** kinetics of the Trp emission band of L18W-PGLa upon partitioning into the lipid bilayer. The plot shows an example at $[P] = 4\ \mu$M and POPE/POPG 3:1 total lipid concentration $[L] = 100\ \mu$M. **B:** best fit (solid red line) of the spectrum after 60 minutes of incubation at $[P] = 4\ \mu$M and $[L] = 100\ \mu$M [see (17) for details]. The curve is well described by the sum of the emission band from PGLa in the aqueous phase (dashed green line) and in the lipid phase (dotted blue line).

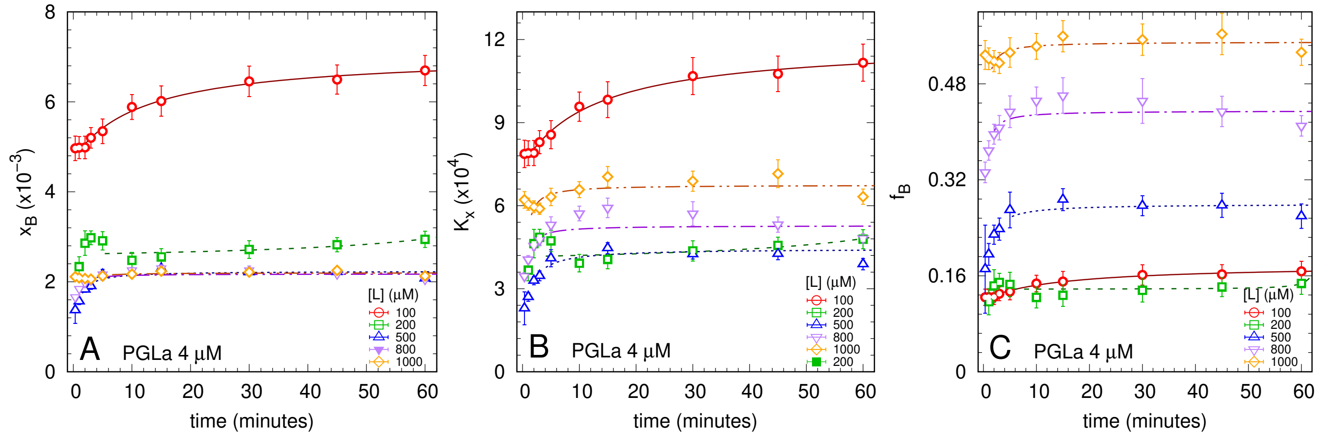

Figure S16: Kinetics of **A:** R_B, **B:** K and **C:** f_B at different POPE/POPG 3:1 concentrations and fixed L18W-PGLa $[P]$ = 4 μM. The lines are just guides for the eyes. Every combination reaches a plateau within < 10 minutes, with the exception of the lower $[L]$ tested (100 μM), where partitioning parameters slowly plateau in the course of about 1 hour.
