# Supplementary material for: Magainin 2 and PGLa in bacterial membrane mimics IV: Membrane curvature and partitioning
Source: Biophys J. 2022 Oct 18;121(23):4689–701. doi: 10.1016/j.bpj.2022.10.018 (PMC9748257; doi:10.1016/j.bpj.2022.10.018)
Supplement: Document S2. Article plus supporting material [file mmc2.pdf]

Biophysical Journal
Article

Biophysical Society

Magainin 2 and PGLa in bacterial membrane mimics IV: Membrane curvature and partitioning

Enrico F. Semeraro,[1,2] Peter Pajtinka,[3,4,5] Lisa Marx,[1,2] Ivo Kabelka,[3,4] Regina Leber,[1,2] Karl Lohner,[1,2] Robert Vácha,[3,4,5,*] and Georg Pabst[1,2,*]

[1]University of Graz, Institute of Molecular Biosciences, Biophysics Division, NAWI Graz, Graz, Austria; [2]BioTechMed Graz, Graz, Austria; [3]CEITEC – Central European Institute of Technology, Masaryk University, Brno, Czech Republic; [4]National Centre for Biomolecular Research, Faculty of Science, Masaryk University, Brno, Czech Republic; and [5]Department of Condensed Matter Physics, Faculty of Science, Masaryk University, Brno, Czech Republic

ABSTRACT We previously reported that the synergistically enhanced antimicrobial activity of magainin 2 (MG2a) and PGLa is related to membrane adhesion and fusion. Here, we demonstrate that equimolar mixtures of MG2a and L18W-PGLa induce positive monolayer curvature stress and sense, at the same time, positive mean and Gaussian bilayer curvatures already at low amounts of bound peptide. The combination of both abilities—membrane curvature sensing and inducing—is most likely the base for the synergistically enhanced peptide activity. In addition, our coarse-grained simulations suggest that fusion stalks are promoted by decreasing the free-energy barrier for their formation rather than by stabilizing their shape. We also interrogated peptide partitioning as a function of lipid and peptide concentration using tryptophan fluorescence spectroscopy and peptide-induced leakage of dyes from lipid vesicles. In agreement with a previous report, we find increased membrane partitioning of L18W-PGLa in the presence of MG2a. However, this effect does not prevail to lipid concentrations higher than 1 mM, above which all peptides associate with the lipid bilayers. This implies that synergistic effects of MG2a and L18W-PGLa in previously reported experiments with lipid concentrations >1 mM are due to peptide-induced membrane remodeling and not their specific membrane partitioning.

SIGNIFICANCE Building on previous reports, we show that MG2a and L18W-PGLa peptides can synergistically destabilize lipid membranes by inducing positive monolayer curvature stress. This effect can be further amplified by the peptides' preferential localization in membrane regions with positive curvature. We also demonstrate that the equiactivity vesicle leakage assay provides the means to connect experimental and computational data to peptide-induced membrane remodeling across a wide range of lipid and peptide concentrations.

INTRODUCTION

The synergistically enhanced activity of magainin 2 and PGLa, two antimicrobial peptides (AMPs) derived from the African clawed frog, at equimolar ratios is well documented both in bacteria and lipid-only model systems (1–4). Some groups, including us, used L18W-PGLa and amidated magainin 2 (MG2a) instead, with similar overall synergistic activity (1,5). Yet, attempts to elucidate the underlying biophysical mechanisms have been only partially conclusive and often led to significant controversies. In the first report on this subject, dye-leakage experiments on lipid vesicles composed of egg yolk phosphatidylglycerol (PG) and phosphatidylcholine (PC) were interpreted via synergistic formation of toroidal pores by MG2a and PGLa (1). This study was followed up by solid-state NMR experiments showing that MG2a and PGLa orientation with respect to the plane of the lipid bilayer varies with membrane composition (6,7). While MG2a was tilted and PGLa adopted a transmembrane orientation for a dimyristoyl PC:dimyristoyl PG mixture (7), both peptides were adsorbed and surface aligned in palmitoyl oleoyl PC:palmitoyl oleoyl PG (POPG) and palmitoyl oleoyl phosphatidylethanolamine (POPE):POPG mixtures, with PGLa slightly tilting into the lipid bilayer (6,7). Clearly, a membrane surface alignment is not in line with the initially proposed

Submitted September 7, 2022, and accepted for publication October 12, 2022.

*Correspondence: robert.vacha@muni.cz or georg.pabst@uni-graz.at

Enrico F. Semeraro and Peter Pajtinka contributed equally to this work.

Editor: Antje Pokorny Almeida.

https://doi.org/10.1016/j.bpj.2022.10.018

© 2022 Biophysical Society.

This is an open access article under the CC BY license (http://creativecommons.org/licenses/by/4.0/).

formation of PGLa:MG2a toroidal pores. Yet, MG2a and L18W-PGLa were also demonstrated to behave synergistically in POPE:POPG (3:1 mol:mol) mixtures, a lipid composition that was found to capture well the activity of both peptides in *Escherichia coli* (5).

Focusing on POPE:POPG (3:1 mol:mol), a follow-up study from our laboratories showed that the two frog peptides preferentially form surface-aligned heterodimers upon membrane adsorption already at low peptide concentrations (8). At high peptide concentrations, in turn, we reported subsequently the formation of fibril-like peptide aggregates sandwiched between lipid multibilayers coexisting with a sponge phase (9). The formation of a sponge phase suggests that the two peptides induce membrane curvature and fusion. Indeed, our molecular dynamics (MD) simulations demonstrated the formation of fusion stalks between the bilayers (9). However, the peptides' induced and preferred curvatures remained unknown.

Most recently we showed that the underlying topological membrane changes, involving vesicle adhesion, fusion, and rupture, occur within a few seconds after peptide addition (10). All three studies assumed that both peptides fully partition into the lipid membranes. This seems to be contrasted by the finite partitioning coefficients reported for fluorescently labeled PGLa and MG2a for the same lipid mixture (11). Interestingly, a significant increase in membrane partitioning of both peptides was observed when applied as an equimolar mixture. This led the authors to suggest that the synergistic activity of PGLa and MG2a is related to an increased membrane affinity. Yet, one of the most fundamental insights from the theoretical framework of membrane partitioning is that the number of membrane-associated peptides depends not only on peptide concentration but also on lipid concentration (12). Therefore, the specific membrane partitioning of PGLa and MG2a reported at low lipid concentrations (11) cannot be directly extrapolated to effects on membrane structure observed at much higher lipid concentrations (8–10).

In the present work, we couple the partitioning behavior of L18W-PGLa in the presence and absence of MG2a in POPE:POPG (3:1 mol:mol) bilayers to dye-leakage experiments. This provides us with the means to scale the response of the membranes to the AMPs according to the used lipid concentration, employing the equiactivity approach (13). As a central finding of these experiments, we demonstrate that synergistically enhanced partitioning of MG2a and L18W-PGLa does not prevail to conditions used in small-angle X-ray scattering (SAXS) experiments. Thus, peptide-induced membrane remodeling reported earlier (8–10) is a result of fully adsorbed/inserted AMPs only. Moreover, results from scattering experiments can be directly related to MD simulations on the same lipid mixture with all peptides associated to the bilayer. This allowed us to focus additionally on the ability of L18W-PGLa and MG2a to synergistically induce and sense membrane curvature, an aspect suggested in our previous studies (9,10). In general, the ability to sense membrane curvature leads to peptide sorting toward the area with the preferred curvature, while curvature induction is the ability of peptides to increase or generate curvature in membranes (14–16).

MATERIALS AND METHODS

Sample preparation

Large unilamellar vesicles

POPG and POPE were obtained from Avanti Polar Lipids (Alabaster, AL, USA) in the form of dry powder. 8-aminonaphthalene-1,3,6-trisulfonic acid, disodium salt (ANTS) and p-xylene-bis-pyridinium bromide (DPX) were purchased from Molecular Probes (Eugene, OR, USA) and HEPES and NaCl from Carl Roth (Karlsruhe, Germany). L18W-PGLa and MG2a were obtained in lyophilized form (purity >95%) from PolyPeptide Laboratories (San Diego, CA, USA); see (5) for the primary peptide structure. Triton X-100 and all other chemicals (proanalysis quality) were obtained from Sigma-Aldrich (Vienna, Austria).

Large unilamellar vesicles (LUVs; size of ∼100 nm) composed of POPE:POPG in HEPES-buffered saline (HBS) solution (10 mM HEPES, 140 mM NaCl [pH 7.4]) were either filled with ANTS/DPX or not were prepared, as detailed previously (5). Unless stated differently, the molar ratio of POPE:POPG used throughout the present study is 3:1 mol:mol.

Peptide partitioning

Tryptophan fluorescence

Tryptophan (Trp) fluorescence emission from L18W-PGLa was measured both in the presence and absence of MG2a using the Cary Eclipse Fluorescence Spectrophotometer (Varian/Agilent Technologies, Palo Alto, CA, USA) at an excitation wavelength of $\lambda = 280$ nm and a slit width of 10 nm for the incident and outgoing beams. Emission spectra were background subtracted to remove contributions from vesicles and the instrument's baseline. All samples were measured at 37°C using a quartz cuvette with a magnetic stirrer to prevent sedimentation. The recorded fluorescence signal was equilibrating for 1 h (Fig. S16); spectra were analyzed as detailed in (17).

In brief, we linearly combined log-normal-like functions (18,19) representing the independent bands for peptides located either in an aqueous environment or partitioned into the lipid bilayer at equilibrium. The wavelength at maximum emission, $\lambda^W = 354.6$ nm, and bandwidth, $\Gamma^W = 64.3$ nm, of L18W-PGLa dissolved in HBS were determined first and fixed for further analysis; both values correspond to Trp exposed to a polar environment (20). The concentration of membrane-dissociated L18W-PGLa in the presence of POPE:POPG, $[P]_W$, was subsequently determined by adjusting the parameters related to membrane-partitioned L18W-PGLa (amplitude, wavelength at emission maximum, width) and scaling the amplitude of the water-associated emission band. The molar concentration of membrane-associated L18W-PGLa is then simply derived from $[P]_B = [P] - [P]_W$, where $[P]$ is the total L18W-PGLa concentration in the sample. This allows us to derive the mole fraction partitioning coefficient

$$K_x = \frac{x_B}{x_W} \simeq \frac{[P]_B}{[L]} \frac{[W]}{[P]_W}, \quad (1)$$

where x_B and x_W are, respectively, the mole fraction of membrane-partitioned L18W-PGLa and free L18W-PGLa in the aqueous environment

(12,21). $[W]$ is the concentration of bulk water (55.3 M at 37°C), and $[L]$ is the concentration of lipids.

The chemical potential of the peptides in either phase $(i = B, W)$ is by definition

$$\mu_i = \mu_i^0 + k_B T \ln(a_i) = \mu_i^0 + k_B T \ln(\gamma_i x_i), \qquad (2)$$

where μ_i^0 is the chemical potential in the standard state, k_B is Boltzmann's constant, T is the temperature, and a_i is the peptide activity in the i-phase (12) (nota bene: the use of k_B rather than the molar gas constant means that μ_i is the chemical potential per single peptide). Finally, the activity can be expressed as product of the molar ratio and the activity coefficient γ_i. At equilibrium, the chemical potentials in both phases have to be the same, $\mu_B = \mu_W$. Hence, the standard free energy of transfer of a single peptide from the water to the lipid phase is written as

$$\Delta G_x^0 = \mu_B^0 - \mu_W^0 \simeq -k_B T \ln\left(\frac{\gamma_B x_B}{x_W}\right) = = -k_B T \ln(K_x) - k_B T \ln(\gamma_B), \qquad (3)$$

where, given the very low peptide concentration in bulk, we approximate $\gamma_w \simeq 1$. In the regime of infinite dilution of partitioned peptides, $(\gamma_B \approx 1)$, which here defines the standard state; ΔG_x^0 is directly obtained from an x_B-independent K_x value (12). In general, however, the peptide activity cannot be neglected. We thus report

$$\Xi := \Delta G_x^0 + k_B T \ln(\gamma_B) = -k_B T \ln(K_x), \qquad (4)$$

which is the free-energy change due to peptide partitioning and membrane activity (aggregation, membrane thinning, etc.).

Leakage assay

Peptide-induced leakage of ANTS/DPX was determined as detailed in (17) over a range of lipid ($[L]$ = 50, 150, 300, and 600 μM) and L18W-PGLa concentrations ($[P]$ = 1–24 μM). Specifically, LUVs were incubated with peptide at 37°C for 1 h using a gently rocking shaker (Eppendorf Thermomixer C, Hamburg, Germany) and then diluted with HBS to a final lipid concentration of 50 μM and sample volume of 2 mL. Measurements were conducted in quartz cuvettes at an excitation wavelength of λ = 360 nm, and emission was recorded at λ = 530 nm, with a slit width of 10 nm for both excitation and emission monochromators, on a Cary Eclipse Fluorescence Spectrophotometer (Varian/Agilent Technologies).

The percentage of leakage, $E_\%$, was calculated using

$$E_\% = \frac{I_p - I_{\min}}{I_{\max} - I_{\min}}, \qquad (5)$$

where $I_{\min}$ is the fluorescence of vesicles measured without peptide and $I_{\max}$ is the fluorescence corresponding to complete leakage after addition of a 1 vol % solution of Triton X-100. The obtained leakage data were interpolated with sigmoidal functions (see Fig. S2), which allowed us to associate pairs of peptide and lipid concentration to specific $E_\%$ values. Then, the partitioning parameters, leading to a specific dye leakage, are given by (13,17)

$$[P] = \underbrace{\frac{x_B^L [W]}{K_x^L}}_{[P]_W} + \underbrace{x_B^L [L]}_{[P]_B} = x_B^L \left(\frac{[W]}{K_x^L} + [L]\right), \qquad (6)$$

allowing the determination of x_B^L and K_x^L as a function of $E_\%$ from linear regressions. The definitions of x_B^L and K_x^L are identical to x_B and K_x. However, x_B^L and K_x^L are apparent observables requiring a change of the physical membrane state (permeability) for detection, while x_B and K_x are only related to peptide partitioning, without "knowing" the associated changes in membrane structure. We therefore prefer to distinguish between them by adding the superscript label L.

Dynamic light scattering

Particle-size distributions and polydispersity of LUVs with and without peptides were determined via dynamic light scattering using a Zetasizer NANO ZSP (Malvern Panalytical, Malvern, UK) equipped with a 10 mW laser (λ = 632.8 nm); scattering was detected at an angle of 173°. All measurements were conducted at room temperature, with 1 mL of sample in quartz cuvettes. Peptide containing systems were incubated at 37°C for 1 h prior to measurements. Data were averaged over 3 scans with 13–15 runs each.

MD simulations

MD simulations were performed using GROMACS, v. 2016.2 (22,23) unless stated otherwise. We employed the MARTINI 2.2 force field (24–26), which has been shown to capture trends in the sensed and induced curvature by proteins (27). The simulation time step was set to 20 fs. A constant temperature of 310 K was maintained via a velocity-rescaling thermostat (modified with a stochastic term) (28) with a coupling constant of 1.0 ps. For proper temperature distribution, two separate baths were coupled to protein-lipid and solvent beads. The pressure was kept at 1 bar using the Parrinello-Rahman barostat (29,30) with a semi-isotropic coupling scheme and a coupling constant of 12 ps. All nonbonded interactions, including van der Waals forces, were cut off at 1.1 nm. The relative dielectric constant was set to 15.

Due to the coarse graining and resulting inability of the MARTINI force field to fold proteins, a fully α-helical secondary structure was imposed on the peptides throughout the entire simulation run. For MG2a, this agrees well with circular dichroism spectroscopy interacting with POPE:POPG (4:1 mol:mol) vesicles (31). However, the helical content of PGLa is known to vary with lipid compositions and can decrease to a 72% helicity in phosphophosphatidylcholine (32). To test if the α-helical content of the peptides affects our results, we performed an independent set of simulations with peptides having lower helical content following previous studies (32,33). For MG2a, a helical backbone structure between residues nos. 4 and 20 was reported (33), while the remaining residues were assigned a coil-like secondary structure. For PGLa, the helix spanned between residues 6 and 21 (32), leaving the N-terminus unstructured. The peptide C-terminal capping was modeled by removing the backbone bead charge and changing the bead type to neutral. All MD simulations were performed exclusively with POPE:POPG (3:1 mol:mol) membranes.

Peptide-induced membrane curvature stress

A symmetric bilayer with a random in-plane distribution of 184 lipids was prepared using the CHARMM-GUI web server (34). The initial box dimensions were 7.5 × 7.5 × 11 nm. Roughly 16 water beads were added per lipid molecule, and Na^+ and Cl^- ions were added at a concentration of 130 mM with excess ions to neutralize the net charge of the system. MG2a and L18W-PGLa were added to prepare the following systems: peptide monomers, parallel peptide heterodimers, and antiparallel peptide homodimers. A copy of either monomer or dimer was placed just above the lipid headgroups in both leaflets having the helical peptide axis aligned parallel to the membrane surface. The total simulation length was 20 μs, and simulation snapshots were saved every 50 ps for membrane-pressure analysis.

Peptide-induced membrane curvature stress was derived by calculating the monolayer lateral stress profiles, $\sigma(z)$, using the Goetz-Lipowsky force decomposition (35) in the modified version of GROMACS 4.5.5 (36,37). In particular, we evaluated the first moment (mean torque), τ, of the lateral stress profile

$$\tau = -\int_0^\infty z\sigma(z)\mathrm{d}z = \kappa_\mathrm{m} c_0^\mathrm{m} \tag{7}$$

for each lipid monolayer in the presence or absence of peptide; z represents the distance from the bilayer midplane, κ_m is the monolayer bending modulus, and c_0^m is the monolayer spontaneous curvature (15,38).

Curvature sensing by peptides

To test the curvature preference of MG2a and L18W-PGLa, buckled and "egg box"-shaped lipid bilayer topologies (with regions of constant nonzero Gaussian curvature K_G) were prepared (Fig. 1). The membrane buckle was shown previously to be a suitable model system for the curvature sensing of large transmembrane proteins (39,40) and small amphipathic helices (41). Using CHARMM-GUI (34), a lipid bilayer was assembled in the XY plane from 1,008 POPE:POPG lipid molecules. The initial box dimensions were 25 × 12.5 × 16 nm. Roughly 30 water beads were added per lipid molecule; Na^+ and Cl^- ions were added at a concentration of 130 mM with excess ions to neutralize the net charge of the system. The curved membrane was created by a compression of the simulation box along the X axis. The extent of membrane compression is quantified in terms of the compressive strain $\varepsilon = (L_{x,0} - L_x)/L_{x,0}$, where $L_{x,0}$ and L_x denote the size of the box along the X axis before and after compression, respectively. Here, we applied $\varepsilon = 0.13$, which is lower than in a previous curvature sensing study ($\varepsilon = 0.2$) (41). Subsequently, the curvature of the membrane was maintained during production runs by fixing the box dimensions in the XY plane, which kept the overall buckled shape of the membrane but allowed it to thermally fluctuate. The system size in the Z direction was allowed to change with an applied pressure of 1 atm. Finally, either one peptide (MG2a or L18W-PGLa), one antiparallel homodimer, or one parallel heterodimer was added to each lipid leaflet. To prevent any bias arising from the initial peptide placement, the peptides were oriented in one leaflet in parallel to the rim of the buckle and orthogonal to this direction in the other leaflet. Each system was simulated twice, with peptides initially positioned in the region of the highest positive or negative curvature (see Fig. S1). The different initial configurations enabled us to verify that our results were independent of initial configurations after 10 μs.

To create an "egg box"-shaped bilayer, a larger membrane patch, with initial box dimensions of 26.3 × 26.3 × 16.9 nm, was created. These simulations were performed with GROMACS v. 2021.2 due to the need of applying a biasing potential

$$\Psi = F_c\{A[\sin(x')\sin(y')] - z_c\}^2 \tag{8}$$

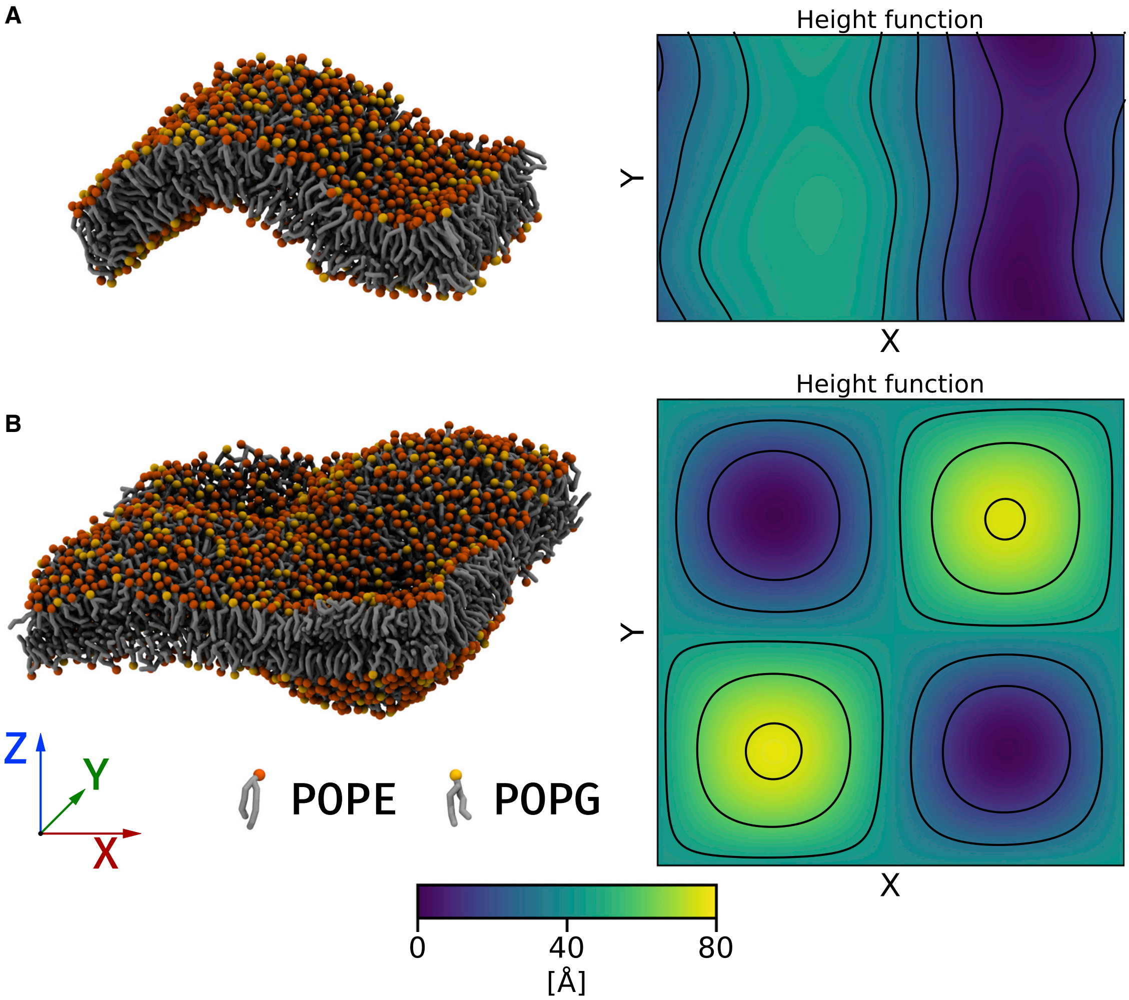

FIGURE 1 Snapshots of the employed model bilayer systems together with the corresponding height function maps illustrating the membrane shape. (*A*) A buckled bilayer system. (*B*) An "egg box" bilayer. Phosphate beads are shown as orange spheres. Glycerol and lipid tail beads are depicted in gray. Solvent, ion, and protein beads are omitted for clarity. The color bar is applicable to maps of both systems. To see this figure in color, go online.

on all phosphate beads in the bottom membrane leaflet. The potential modified the phosphate position along the Z axis (membrane normal) depending on their x,y position. F_c is the force constant, A is the undulation amplitude, x' and y' are scaled coordinates (in the range of $[-\pi, \pi]$) of a phosphate bead relative to the box center, and z_c is the reference displacement from the center of mass of phosphate beads in the lower membrane leaflet. After equilibrating a flat membrane for ~20 ns, the curvature was generated over the course of 25 ns by gradually increasing F_c to 5 kJ mol^{-1} nm^{-2}. We simulated each system (no peptides, peptide monomers/dimers) starting from two different initial conditions. For systems with MG2a and L18W-PGLa peptide monomers, one peptide was placed on each membrane leaflet with a random initial position. Three different amplitudes of the curved surfaces ($A = 1, 2.5$, and 4 nm) were studied. In systems with preformed homo- and heterodimers, two dimers were placed on each membrane leaflet of an already curved membrane: one in a region with the most positive and one in a region with the most negative Gaussian curvature. The amplitude in these systems was set to 2.5 nm. Simulated systems are summarised in Table S1.

To evaluate the curvature preference of the studied peptides quantitatively, we employed an approach previously used in the study of Bhaskara et al. (39). In this approach, the membrane shape is approximated by the positions of phosphate (PO_4) lipid beads, which were fitted using a two-dimensional Fourier series to obtain a differentiable curved surface. From this continuous surface, we calculated local membrane curvature at the point corresponding to the peptide center of mass. For comparison, peptides forming dimers were also evaluated in their monomeric state. From the two principal curvatures obtained, c_1 and c_2, we calculated the mean, $H = \frac{1}{2}(c_1 + c_2)$, and Gaussian curvatures, $K_G = c_1 c_2$. In contrast to the original study of Bhaskara et al. (39), we fitted each membrane leaflet individually. The first 5 μs of the buckled membrane trajectories were omitted from analysis and discarded as equilibration. Due to high computational costs, only the first 1.5 μs were discarded in the case of “egg box” bilayer systems. Note that only the peptides in the upper leaflet, i.e., not directly experiencing the biasing potential applied to the bottom leaflet, were analyzed.

RESULTS

Partitioning of L18W-PGLa depends on peptide and lipid concentration and is enhanced in the presence of MG2a

We first investigated the partitioning of L18W-PGLa into the POPE:POPG mixture using Trp fluorescence at $[P] = 4$ μM as a function of total lipid concentration ($[L] = (100 - 1{,}000$ μM). The fluorescence signal from the partitioned AMPs exhibited a blue shift upon addition of the peptide, with values of $\lambda^B \simeq (330 - 333)$ and $\Gamma^B \simeq (50 - 53)$ nm. This provides evidence for an average location of the Trp residue of L18W-PGLa within the hydrophobic region of the lipid bilayer (20). Applying the peptide partitioning analysis, detailed in the materials and methods section, we derived the dependencies of the number of membrane-partitioned peptide per lipid, x_B, the mole fraction partitioning coefficient K_x, and the fraction of partitioned L18W-PGLa $f_B = [P]_B/[P]$ on lipid concentration (Fig. 2, *A–C*).

By increasing the lipid concentration, we observed a rapid decrease of x_B for $[L] \leq 200$ μM to about 500 lipids per partitioned peptide (Fig. 2 *A*), while the fraction of partitioned L18W-PGLa increased strongly at higher lipid concentrations (Fig. 2 *C*). The partitioning coefficient, in turn, showed a pronounced minimum at $[L]^* \sim 500$ μM (Fig. 2 *B*), corresponding to a local maximum of $\Xi = -10.5$ k_BT and concurring with the leveling off of x_B. This value signifies that the number of membrane-associated peptides does not increase for $[L] > [L]^*$ despite either energetically more favorable peptide partitioning or increased peptide activity. This is probably due to the fact that, despite the ~twofold increase of K_x, the actual energy gain of $\Delta\Xi \sim -0.6$ k_BT between $[L]^*$, and the maximum measured lipid concentration is low and dissipates within thermal “noise”; see the discussion section for further interpretation of these results.

In the next step, we fixed the lipid concentration at the lowest level ($[L] = 100$ μM) and studied the partitioning thermodynamics of L18W-PGLa as a function of peptide concentration, both in the absence and presence of MG2a (Fig. 2, *D–F*). For both scenarios, we observed a monotonous increase of x_B, albeit the effect was up to about four times more pronounced in case of the equimolar mixture with MG2a. The partitioning coefficient K_x of L18W-PGLa, calculated via Eq. 1, in turn showed a maximum, and its general trend was about three to four times higher when MG2a was present. This implies that there is either a gain in energy of transfer or an enhanced peptide activity—or a combination of both—by moving L18W-PGLa from the bulk to the lipid phase in case of the equimolar mixture. However, the energy difference between the two K_x maxima is less than $\Delta\Xi \sim -1.2$ k_BT, showing that there is little free-energy gain upon the addition of MG2a. Further, despite the overall higher K_x values in the case of the peptide mixture, the x_B values in the absence and presence of MG2a are comparable when the total peptide concentration is about 2 μM. Finally, the fraction of membrane-associated L18W-PGLa is roughly doubled in the case of the equimolar mixture, $f_B \sim 0.3 - 0.4$ (Fig. 2 *F*), independent of the total peptide concentration.

In order to correlate the partitioning of L18W-PGLa with its activity, we performed dye-leakage experiments according to the “equiactivity” approach introduced by Heerklotz and Seelig (13); see also (17). Unlike our previously reported leakage experiments with the same lipid mixtures and peptides (5), where we titrated the peptides at a given lipid concentration, the present experiments were performed over a range of lipid and peptide concentrations using the protocol detailed in the materials and methods section; raw leakage data are shown in Fig. S2. Interpolation of these data in terms of a sigmoidal function allowed us to retrieve partitioning parameters (Eq. 6) for defined dye-leakage values (Fig. 3 *A*). x_B^L showed a sigmoidal increase, reaching a value of ~0.025 at nearly 100% leakage (Fig. 3 *B*). K_x^L, in turn, decreased monotonously as a function of leakage percentage (Fig. 3 *C*), i.e., with increasing $[P]$ but independent of $[L]$.

We additionally performed dynamic light scattering measurements 1 h after incubation with the peptides in order to

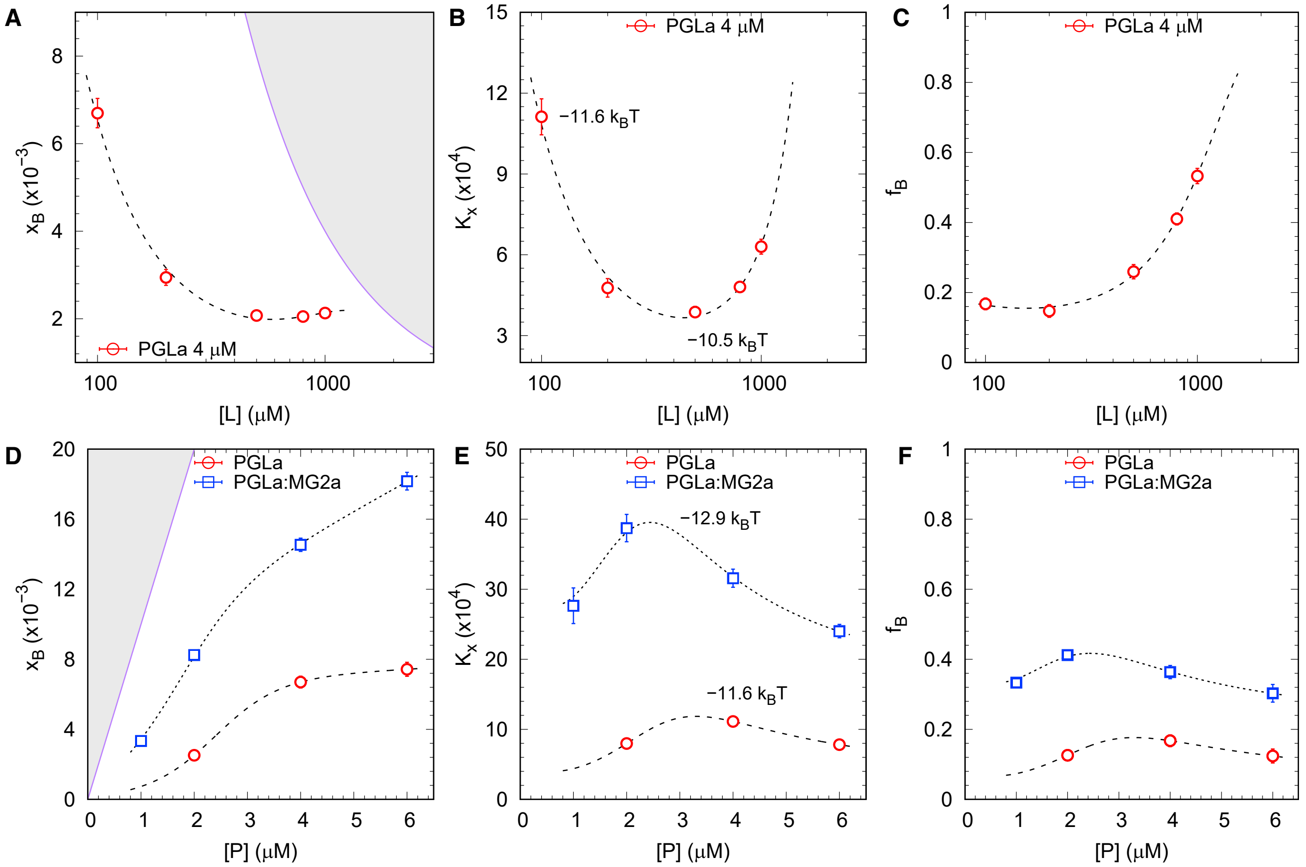

FIGURE 2 Partitioning of L18W-PGLa in POPE:POPG LUVs from Trp fluorescence experiments. (*A*) Number of partitioned L18W-PGLa per lipid molecule, and (*B*) partitioning coefficient and (*C*) fraction of partitioned peptide as a function of $[L]$ at $[P] = 4$ μM. (*D–F*) Plots of x_B, K_x, and f_B as a function of L18W-PGLa concentration, $[P] \equiv [\text{PGLa}]$, at $[L] = 100$ μM for L18W-PGLa in the absence and presence of MG2a (1:1 mol:mol). Corresponding $\Xi = -k_B T \ln(K_x)$ values are given for selected lipid and peptide concentrations. Hashed regions in (*A*) and (*D*) indicate inaccessible values, i.e., $x_B > [P]/[L]$. Dashed lines are guides for the eyes that obey physical constraints (including $x_B \leq [P]/[L]$ and $\lim_{[P]\to 0} x_B = 0$). Trends shown in (*B*), (*C*), (*E*), and (*F*) are calculated from those shown in (*A*) and (*D*). To see this figure in color, go online.

understand if the observed changes of partitioning are potentially correlated to morphological changes of the LUVs. These measurements were performed at the same lipid and peptide concentrations that we explored in Trp fluorescence experiments. In addition, MG2a alone systems were measured at $[L] = 100$ μM and $[P] = 2$, 4, 8, and 12 μM. No significant changes in LUV size or size distribution were observed upon adding either L18W-PGLa or MG2a alone (Fig. S3). In contrast, the equimolar peptide mixture first led to a significant broadening of the size distribution but also to increased hydrodynamic radii, indicating the formation of large lipid/peptide aggregates.

MG2a and L18W-PGLa induce and sense membrane curvature

We used MD simulations to calculate if the membrane adsorption of either peptide induces curvature stress in membrane leaflets. Such a stress could lead to the formation of either positively or negatively curved membrane surfaces and cause membrane fusion through the formation of a fusion stalk (9) or pores. In particular, we simulated a flat membrane with and without peptides and calculated the corresponding lateral stress profiles as detailed in the materials and methods section.

Fig. 4 *A* shows the lateral stress profile in one of the POPE:POPG leaflets. The maximum stress occurs at the polar/apolar interface and is a result of the interaction between the hydrophobic core and water (42). This positive stress peak is accompanied on both sides by negative stresses corresponding to interactions between lipid headgroups and interactions between hydrophobic chains, respectively (43,44). Calculating the mean torque (Eq. 7) and using $\kappa_m = 15.2 \pm 0.7$ k$_B$T, obtained from a weighted average of POPE and POPG (45), we arrive at $c_0^m = -0.320 \pm 0.015$ nm for the monolayer spontaneous curvature of POPE:POPG. This spontaneous monolayer curvature is slightly more negative but is still in reasonable agreement with our previously reported experimental estimate, $c_0^m = -0.26 \pm 0.01$ nm (5).

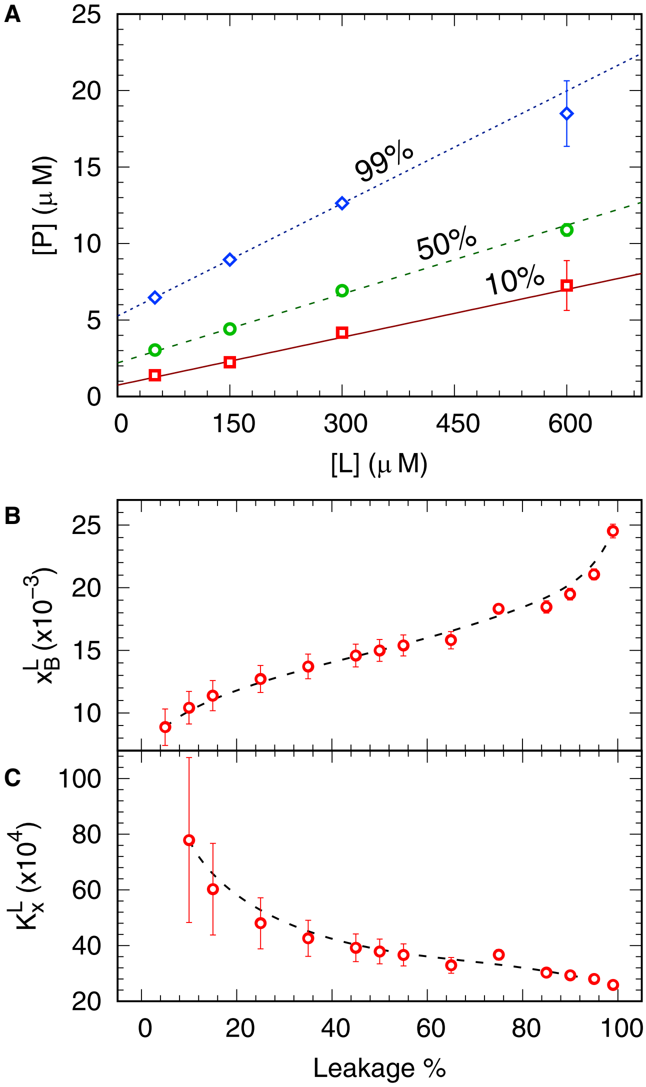

FIGURE 3 Connecting dye leakage to peptide partitioning. (*A*) Selected sets of lipid and L18W-PGLa concentrations, [*P*], leading to 99% (*blue diamonds*), 50% (*green circles*), and 10% (*red squares*) dye leakage. Data were fitted with Eq. 6. Apparent values of partitioned peptide to lipid ratio (*B*) and molar partitioning coefficient (*C*) for L18W-PGla at various degrees of dye leakage. Dashed lines are guides for the eyes. To see this figure in color, go online.

Adding L18W-PGLa and MG2a, either as monomer or dimer, leads to significant changes of the lateral stress profiles (Fig. 4, *B* and *D*). The peptide-induced changes are mainly occurring in the interfacial region of the bilayer, i.e. where the peptides are residing. The positive stress peak at the water-hydrocarbon interface decreased (negative peak in the stress difference plot), and was accompanied by a positive stress increase in the hydrocarbon chain region due to peptide insertion; see also (44). These stress changes were the largest for both L18W-PGLa:MG2a heterodimer and the two homodimers, whereas monomers led to small differences in lateral stress as compared to the peptide-free system only.

The effect of all studied peptides and their combinations on membrane curvature stress can be seen from the induced increase of the mean torque listed in Table 1. Corresponding results for peptides with reduced helicity are listed in Table S2 and show only minor differences. Additionally, we report the changes on spontaneous monolayer curvature, assuming that κ_m remains constant (see Eq. 7). All reported values in Tables 1 and S2 are averaged over both symmetric lipid leaflets in the membrane. Previous experimental studies reported a decrease of κ_m in the presence of peptides, see, e.g., (46,47). Consequently, the Δc_0^m s calculated with κ_m from pure membrane are lower limit estimates.

Comparing quantitative changes, one has to bear in mind that the system size was the same leading to a doubling of the $[P]/[L]$ ratio for dimers compared with monomers. For peptide monomers, the effect of MG2a was approximately two times larger than for L18W-PGLa. Among the dimers, MG2a homodimers caused the largest increase in τ, followed by L18W-PGLa:MG2a heterodimers and L18W-PGLa homodimers. The effect of dimers was roughly double that of monomers. However, note that the stability of the dimers varies and that the heterodimer is the most stable among all dimers (8).

Secondly, we addressed also the question whether L18W-PGLa or MG2a are able to sense "preformed" positive or negative membrane curvatures. To do so, we constructed a buckled membrane with regions of both positive and negative curvature (see Fig. S4). The membrane was curved only in one direction (*X* axis) and had a zero average curvature in the second direction (*Y* axis) (see Fig. S1). However, subtle thermal membrane undulations in both directions were observed during the simulation (Fig. S4). We analyzed the preferred position of peptides on the buckled membrane and calculated the corresponding local curvature as detailed in the materials and methods section.

The average values of sampled mean bilayer curvature are shown in Table 2; corresponding histograms are provided in Fig. S5. Again, results obtained for peptides with lower helicity differed only marginally (see Table S4 and Fig. S6). All simulated peptides, the MG2a monomer, the L18W-PGLa monomer, their homodimers, and the L18W-PGLa:MG2a heterodimer sensed positive mean curvature. Overall, dimers exhibited preference for larger positive curvatures than monomers. Within the dimers, the homodimer of MG2a preferred the largest curvature, closely followed by the heterodimer, and the lowest positive curvature was preferred by the L18W-PGLa homodimer. Monomeric MG2a occupied regions of higher mean curvature than the PGLa monomer. Hence, the trend in sensing membrane curvature follows the one for inducing monolayer curvature stress.

For the Gaussian curvature, we obtained a similar preference as for the mean curvature. A detailed look at the probabilities of peptide curvature preferences (Fig. 5) shows that L18W-PGLa and MG2a monomers prefer positive principal

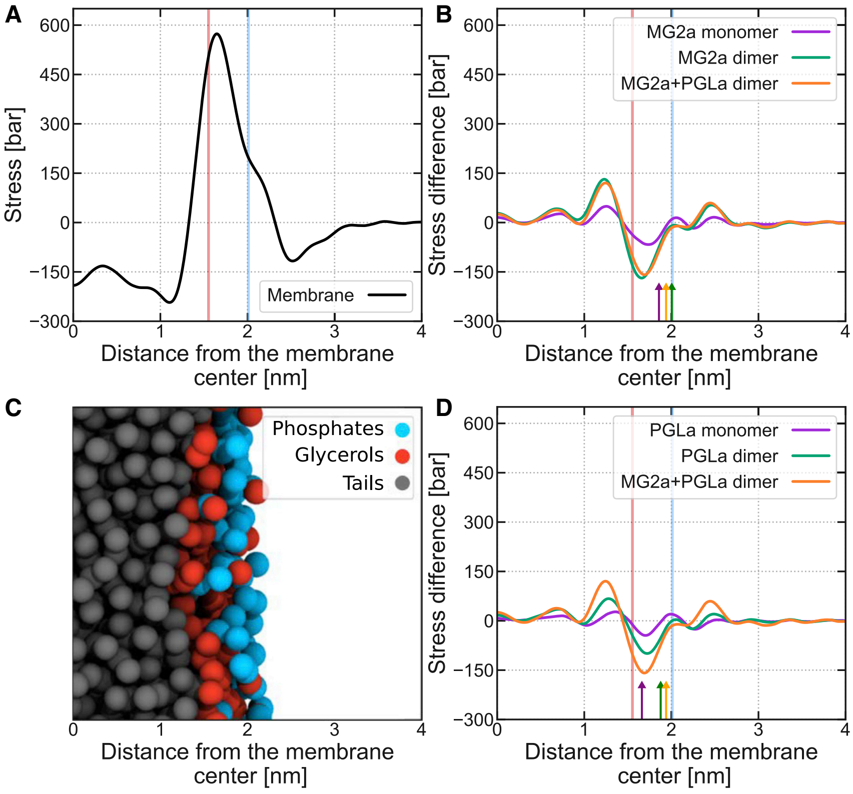

FIGURE 4 Calculated lateral stress profiles for planar membranes, σ, as a function of the distance from the membrane center. (*A*) Reference stress profile for a membrane composed of 3:1 (mol:mol) POPE:POPG lipids. (*B* and *D*) Changes in the stress profiles induced by the presence of (*B*) MG2a and (*D*) L18W-PGLa peptides, together with the changes induced by the heterodimer. In each figure with stress profiles, positions of glycerol (*red*) and phosphate (*blue*) groups are indicated by vertical lines, as depicted in the snapshot in (*C*) (water beads are omitted for clarity). The positions of peptides are indicated by arrows, derived from the maxima of averaged density profiles. To see this figure in color, go online.

curvature values c_1 and c_2. The dimers prefer larger c_1s than monomers, while c_2 is roughly the same. Therefore, changes in c_1 appear to be key to the curvature sensing difference between monomers and dimers.

Furthermore, we investigated if the preference for Gaussian curvatures holds also for membranes with significant curvatures in two directions using an "egg box" bilayer. Despite the different topology the trends of the preferred Gaussian curvature are in agreement with the preferences obtained for buckled membranes. Snapshots of the "egg box" membrane systems together with maps of mean and Gaussian curvatures are provided in Fig. S7. The monomers and the PGLa homodimer sampled a wide range of Gaussian curvatures, while the MG2a homodimer and L18W-PGLa:MG2a heterodimer showed a preference for larger positive Gaussian curvature (Figs. S8–S10). This trend was not affected by the membrane amplitude. However, the displayed preferences were enhanced at higher curvature amplitudes (Fig. S11).

The peptide sensing of high positive curvature might be a secondary effect of enhanced lipid/peptide interactions due to the preference of certain lipids (based on their molecular shape) to accumulate in specific membrane regions. Note that we have previously observed a strong interaction between POPG lipids and both MG2a and PGLa peptides in all-atom simulations (8). Here, we confirmed the POPG preference for all peptides monomers and dimers in MARTINI simulations on a planar membrane (Fig. S12). Subsequently, we compared the lipid distribution on a buckled membrane in the absence, and then in the presence, of the peptides. In agreement with their overall molecular shape, POPG lipids were slightly more populated in membrane regions of positive curvature, while POPE lipids accumulated more in the regions of membrane negative curvature (Fig. S13). However, the preference was subtle, and there were still many POPG lipids in the region of membrane with negative curvature. Therefore, the peptides had the preferred POPG lipids available in all membrane regions and peptides did not significantly alter the overall lipid distribution. The peptide preference for positive curvature thus does not seem to be affected by a small POPG preference for membrane positive curvature.

DISCUSSION

Unraveling the partitioning of any membrane active compound is an essential requisite in understanding its mode of action. In the case of AMPs, many such studies have been performed decades ago (for review see, e.g., (48)). Nevertheless, this is certainly also not sufficient for obtaining a holistic picture of all associated events occurring on the molecular level. To reach this goal, the peptide partitioning needs to be coupled to an experimentally observable effect. In the case of AMPs, fluorescent dye-leakage assays on lipid vesicles are common experiments to screen for the potency of these compounds in interfering with the

TABLE 1 Peptide-induced changes of mean torque, $\Delta\tau$, and spontaneous monolayer curvature, Δc_0^m

Peptides	$\Delta\tau$ [10^{-12}Jm^{-1}]	Δc_0^m [nm^{-1}]
MG2a (monomer)	1.88 ± 0.15	0.029 ± 0.003
L18W-PGLa (monomer)	0.98 ± 0.18	0.015 ± 0.003
2x MG2a (dimer)	3.7 ± 0.6	0.057 ± 0.009
2x L18W-PGLa (dimer)	2.3 ± 0.9	0.036 ± 0.014
L18W-PGLa:MG2a (dimer)	2.75 ± 0.19	0.043 ± 0.004

The values are averaged over both symmetric lipid leaflets in the membrane.

membrane's barrier function (see, e.g., (1,5)). The equiactivity approach put forward by Heerklotz and Seelig (13) allows appropriate scaling of peptide-induced leakage to the lipid concentrations used in other, complementary experiments. Fig. 6 shows such a "leakage-activity map" for the activity of L18W-PGLa in POPE:POPG (3:1 mol:mol) membranes, connecting different experimental and computational tools applied to study PGLa/MG2a synergism.

We performed Trp-fluorescence spectroscopy experiments of L18W-PGLa and L18W-PGLa:MG2a using POPE:POPG LUVs over a range of lipid (100 μM $\leq [L] \leq$ 1 mM) and peptide (1 μM $\leq [P]_{\mathrm{PGLa}} \leq$ 6 μM) concentrations. Trp-emission spectra were fitted with a heuristic model providing a direct measure of L18W-PGLa partitioning parameters. That is, only $[P]_W$ is directly retrieved from the spectra, so one does not need any a priori information about the band parameters of the Trp emission from membrane-associated peptides. This analysis indeed resulted in band width and position values consistent with Trp residues in an apolar environment, but they were not sufficient to distinguish between folding state and/or peptide association. We observed that the partitioning of L18W-PGLa both in the absence and presence of MG2a varied in a nontrivial way with lipid and peptide concentration (Fig. 2). In particular, the nonconstant K_x value provides strong evidence for complex interplay between lipid-peptide and peptide-peptide interactions, i.e., $\gamma_B \neq 1$. In fact, the increase of K_x values (Fig. 2, *B* and *E*) could be attributed to a cooperative membrane association due to peptide oligomerization (12), while decreasing K_x values could refer to a rising electrostatic screening effect for high x_B values (i.e., anticooperative effect (12)). Peptide oligomerization after membrane insertion has been observed both by MD (9) and experimentally (11). The effect on K_x related to electrostatic interactions is likely to occur with charged peptides, and the nonlinear increase of with free peptide, $[P]_W$ (Fig. S14), is a clear signature of this scenario (see (48) for details). The about three- to fourfold higher partitioning coefficient in the presence of MG2a, as well as the more pronounced maximum, is an explicit expression of PGLa/MG2a synergism. Yet, the fraction of membrane-associated L18W-PGLa does not exceed 0.4 even in the presence of MG2a. This signifies that at $[L] = 100\ \mu$M, most of the peptides remain unbound within the buffer.

TABLE 2 Average values of the preferred mean curvature, *H*, by the peptides on the buckled POPE:POPG (3:1 mol:mol) membrane, i.e., sensed curvature

Peptides	H [nm^{-1}][a]
MG2a (monomer)	0.0848 ± 0.0006
L18W-PGLa (monomer)	0.0650 ± 0.0022
MG2a (dimer)[b]	0.1033 ± 0.0003
L18W-PGLa (dimer)	0.0859 ± 0.0004
L18W-PGLa:MG2a (dimer)[b]	0.1017 ± 0.0003

The standard error of the mean was calculated from two independent simulations.

[a]In each system, peptides at individual leaflets were considered independent, resulting in a total number of four independent systems.

[b]One of the dimers dissociated during the simulation run, which was not included in the analysis (for details, see supporting material).

The increased partitioning of L18W-PGLa in the presence of MG2a is consistent with a recent report on the same lipid mixture using a self-quenching assay of fluorescently labeled peptides (11). Converted to the here-used partitioning notation, their apparent partitioning values for PGLa in the absence and presence of MG2 are $K^{\mathrm{app}} = (530 \pm 137) \times 55.3 = (2.9 \pm 0.8) \times 10^4$, and $K^{\mathrm{app}} = (33 \pm 17) \times 10^4$, respectively. Using Eq. 6, at $[L] = 100\ \mu$M, this corresponds to $f_B \sim 0.05$ in the absence of MG2a and $f_B = 0.23 - 0.48$ in the case of the equimolar peptide mixture. These values can be compared with our results (Fig. 2, *E* and *F*), extrapolating to $[P] = 10\ \mu$M (as used in (11)). The lower f_B at higher peptide concentration for L18W-PGLa alone suggests that the affinity for membrane partitioning decreases further at high $[P]$, whereas it remains roughly unchanged in the presence of MG2a. This different behavior could suggest a competition between the increasing electrostatic screening and propensity to form either homo- or heterodimers.

Leakage data were modeled with the equiactivity approach (13) to determine the partitioning coefficients K_x^L and x_B^L (Fig. 3). Fig. 3 *C* shows how K_x^L values decrease as function of dye efflux, i.e., with peptide concentration (Fig. S2). Although, this trend is qualitatively in line with our Trp-fluorescence data at high $[P]$ (Fig. 2, *E*), the absolute values are up to about eight times higher. This might be related in part to the fact that leakage assays probe the irreversible dye efflux caused by the complex interactions between peptide monomers/oligomers and lipid membrane, i.e., peptide insertion and folding, translocation, pore/defect formation, and so forth. Trp fluorescence, in turn, just "counts" peptides in a polar (or apolar) environment.

Despite these complications, the equiactivity analysis (13) is highly valuable for extrapolating a given degree of membrane permeation at a specific ($[L]$, $[P]$) pair to conditions used in other experiments or simulations, thus enabling a comparison of effects (Fig. 6). In particular, we reported the transformation of POPE:POPG LUVs into multilamellar vesicles by either L18W-PGLa and MG2a at $[P]/[L] = 1/25$ (9), while the LUVs remained intact at $[P]/[L] = 1/200$ (8). A comparison with Fig. 6 shows that the topological

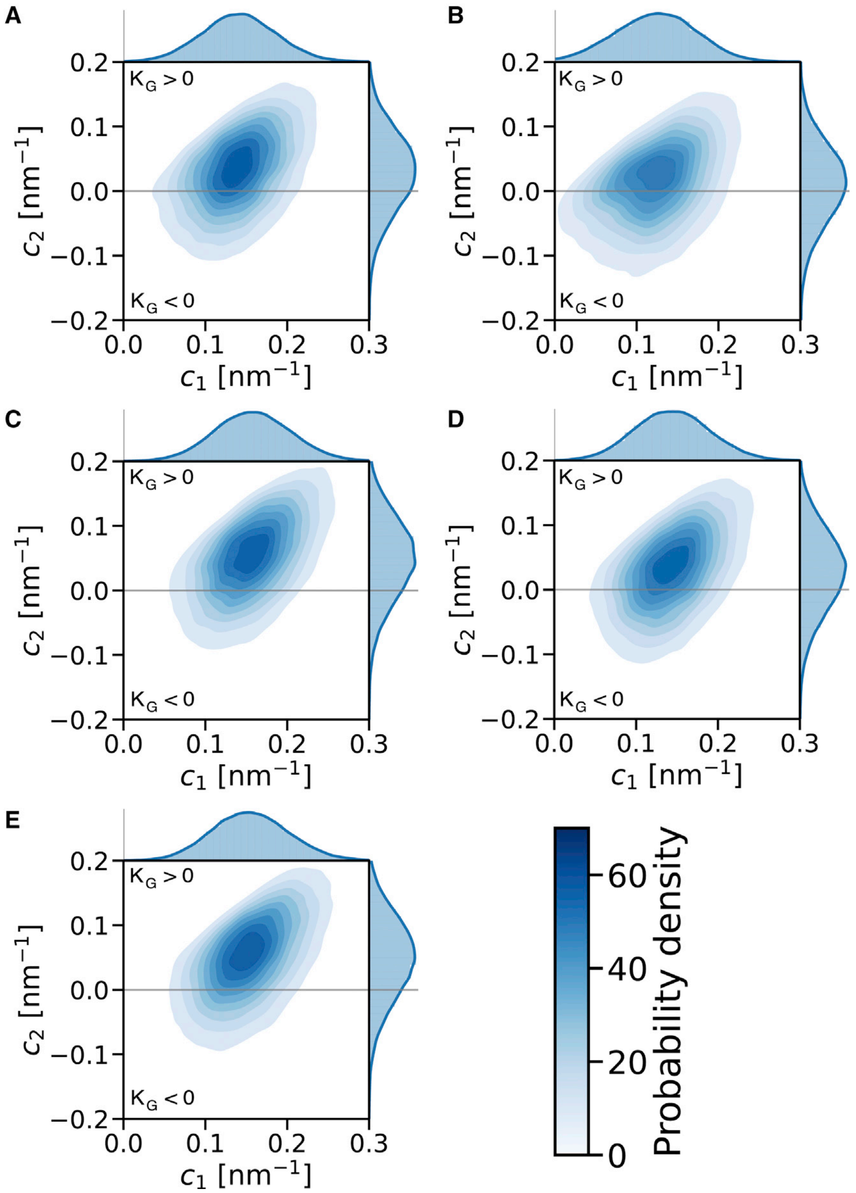

FIGURE 5 Two-dimensional histogram of principal curvatures, c_1 and c_2, sampled by studied peptides on the POPE:POPG (3:1 mol:mol) buckled bilayer. Regions of positive and negative Gaussian curvature, $K_G = c_1c_2$, are divided by gray lines. (*A*) MG2a as a monomer sampled small positive Gaussian curvatures, similar to (*B*) L18W-PGLa monomers and (*C*) MG2a homodimers. (*D*) A homodimer of L18W-PGLa preferred near-zero Gaussian curvatures, observed by a shift of the c_2 peak toward zero curvature. On the contrary, (*E*) the L18W-PGLa:MG2a heterodimer sampled positive Gaussian curvature as evidenced by a shift of c_2 toward positive curvature. To see this figure in color, go online.

transformation at high peptide concentration is, unlike previously conjectured (9), related to enhanced membrane permeation. This further implies that the formation of a sponge phase for L18W-PGLa:MG2a (1:1 mol:mol), which was also observed at high $[L]$ and $[P]$ (9), is a feature of the peptides' synergistic activity but is not uniquely correlated to the enhanced dye leakage reported earlier (5). Moreover, one of the central insights of the partitioning framework is that the fraction of membrane-associated peptide increases with lipid concentration and becomes independent of details of the peptide's amphipathicity (for $[L] \gg [W]/K_x$, see Eq. 6). This implies a negligible amount of unbound peptide at the high lipid concentrations used in SAXS experiments and enables a direct comparison with MD simulations. That is, for $[L] \gg [W]/K_x$, differences between MG2a and L18W-PGLa affinities for POPE:POPG are negligible since $f_B \sim 1$. Note that Fig. 6 also implies that L18W-PGLa:MG2a equimolar mixtures induce membrane permeation, which is comparable to the individual peptides for $[L] \gtrsim 1$ mM, i.e., the synergistic gain is drastically reduced.

From our previous simulations, we reported that equimolar mixtures of L18W-PGLa and MG2a remain surface adsorbed even at a high peptide concentration and aggregate into dimers on membrane and fibril-like structures, sandwiched between bilayers with a collapsed water spacing (9). Moreover, we observed the occasional formation of fusion stalks between adjacent bilayers in simulations, and in experiments, peptides caused formation of sponge phase. These results suggest that the peptides could be able to induce topological membrane changes, which are both connected to leakage and membrane curvature. Here, we

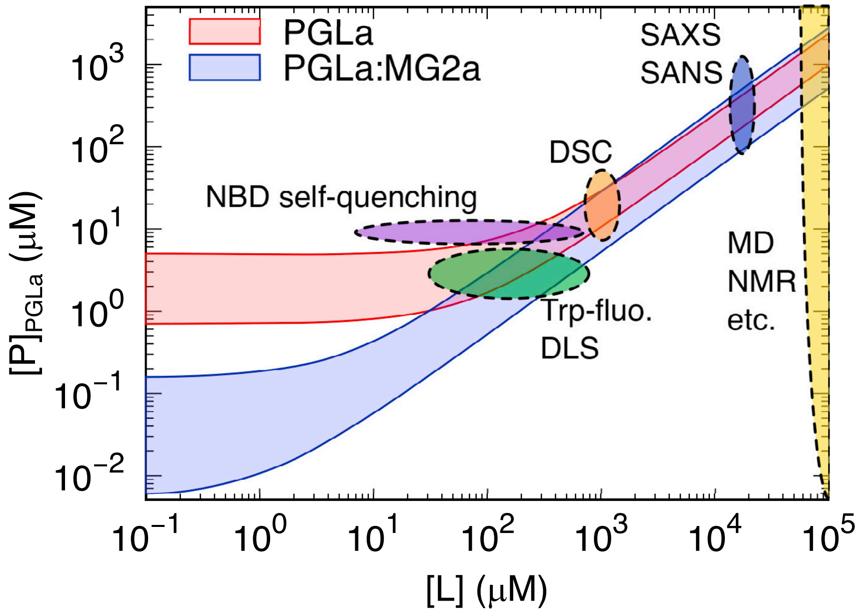

FIGURE 6 Schematic equiactivity map for L18W-PGLa and its equimolar mixture with MG2a based on leakage experiments reported in Fig. 3 *A* and (5) for POPE:POPG (3:1 mol:mol) bilayers. Solid lines enclose ($[L]$; $[P]$) values leading to 10%–99% leakage for L18W-PGLa alone (*red lines*) and L18W-PGLa:MG2a (*blue lines*). The latter are based on present experiments and data reported in (5,11). The map includes lipid and peptide ranges used in various studies on PGLa:MG2a synergism, including the present study with: Nitrobenzoxadiazole (NBD)-based self quenching (11), Trp-fluorescence and dynamic light scattering, differential scanning calorimetry (9), and SAXS/small-angle neutron scattering (8,9). The yellow area is a simplification for all the approaches that assume $f_B \sim 1$ (e.g., MD simulations) or systems at reduced levels of hydration, for which $[L] \gg [W]/K_x$ (e.g., solid-state NMR (6,7)). To see this figure in color, go online.

interrogate whether or not both peptides are either able to induce or sense membrane curvature alone or if this is a signature of their synergism.

Our results provide evidence that L18W-PGLa and MG2a peptides as monomers or homo- and heterodimers not only induce positive monolayer curvature stress but also prefer surface adsorbed states in regions of positive mean and Gaussian bilayer curvatures. The fact that both peptides and their dimers induce positive monolayer curvature stress may seem counterintuitive since both membrane pores and fusion stalks have negative Gaussian curvature (49). However, the peptides could promote formation of such structures by lowering the associated free-energy barrier without having a preference for negative Gaussian curvature. Indeed, we have observed that peptides cause membrane adhesion (local proximity of membranes is the necessary step in the formation of fusion stalk) and that the hydrophobic residues in the middle of dimer act as a hydrophobic bridge between the membranes, which enhances the formation of lipid splay starting the stalk formation (9). In agreement, we also showed that the peptides avoided the membrane stalk region after its formation (10). Note that the obtained preference of peptides for positively curved lipid surfaces is consistent with previous experimental reports on other short linear peptides (50,51).

Comparing the peptides quantitatively, the largest effects were calculated for MG2a monomers and dimers both in terms of curvature induction as well as in curvature sensing. Importantly, these results are consistent even for partially unfolded peptides, and we are able to claim that peptides induce curvature at $[P]/[L] = 1/92$, i.e., at conditions that cause minor leakage (<10%) based on the equiactivity approach, see Fig. 3 *A*. It may come as a surprise that heterodimers of L18W-PGLa and MG2a have a lower efficacy as curvature effectors and sensors than MG2a homodimers. However, we have reported previously that MG2a homodimers are less stable compared with L18W-PGLa:MG2a heterodimers (8). Thus, even if L18W-PGLa:MG2a heterodimers are less effective than MG2a homodimers, the higher tendency to form heterodimers on POPE:POPG bilayers is expected to cause a stronger net curvature stress in bilayers. Note that the overall induced curvature of monomers and dimers is within our calculation error, but the effect of dimers is more localized. Moreover, once the membrane bulges outward, the positive membrane curvatures provide an additional attractor for both peptides. This additionally supports the formation of L18W-PGLa:M2a heterodimers or aggregates of higher number in a self-amplified type of reaction.

Finally, our simulations revealed a preference of L18W-PGLa:MG2a heterodimers (as well as homodimers and individual peptides) for both positive principal curvatures c_1 and c_2 using two independent membrane models and peptides with full and partial helicity (Fig. 5). This corroborates our previous observation that both peptides do not have a preference for fusion necks (10). The formation of a sponge phase (with negative Gaussian curvature), as reported previously (9), might require peptide-mediated membrane adhesion or peptide refolding/different aggregation states. Such a study is beyond the scope of the present work, however.

CONCLUSION

Our direct measurements of peptide partitioning in a POPE:POPG bilayer confirm increased membrane association of L18W-PGLa in the presence of MG2a. However, we also clearly demonstrated that in the case of membrane permeabilization, this difference is significant only at low lipid concentrations. Many of the techniques, which were applied to

study the synergistic activity of L18W-PGLa and MG2a, have an experimental window at $[L] \gg 1$ mM (e.g., SAXS/small-angle neutron scattering) or do not allow for an exchange of the peptide with a bulk aqueous phase (e.g., solid-state NMR or MD simulations). That is, synergistic effects of L18W-PGLa and MG2a observed under these conditions are not affected by their different partitioning parameters. Moreover, the typically high concentration of AMPs in cellular envelopes of live bacteria at minimum inhibitory concentrations (17) suggests that measurements at high $[L]$ mimic natural conditions much more closely. The equiactivity approach entails a viable route to bridge the different experimental and computational techniques. Here, we showed that the energetically stabilized L18W-PGLa:MG2a heterodimer (8) is able to destabilize membranes by inducing a positive mean torque already at relatively low amounts of bound peptide ($[P]/[L] = 1/92$). Additionally, the membrane-adsorbed peptides (both monomers and dimers) also likely accumulate at regions of positive mean and Gaussian curvature with dimers locally enhancing the destabilization effect. Coupling of peptide-induced and sensed curvatures most likely initiates the complete release of dyes encapsulated in vesicles or the formation of a sponge phase, as observed in experiments.

SUPPORTING MATERIAL

Supporting material can be found online at https://doi.org/10.1016/j.bpj.2022.10.018.

AUTHOR CONTRIBUTIONS

E.F.S. analyzed all experimental data. P.P. and I.K. carried out and analyzed all computational simulations. L.M. and R.L. performed experiments. E.F.S., K.L., R.V., and G.P. designed the research. E.F.S., P.P., I.K., R.V., and G.P. wrote the article.

ACKNOWLEDGMENTS

This work was supported by the Austrian Science Fund FWF (project nos. P 30921 and I1763-B21 to K.L.), the Czech Science Foundation (grant 20-20152S to P.P. and I.K.), the European Research Council (ERC) under the European Union's Horizon 2020 research and innovation program (grant agreement no. 101001470 to R.V.), and the National Institute of Virology and Bacteriology (Program EXCELES, ID project no. LX22NPO5103), funded by the European Union - Next Generation EU (to R.V.). Computational resources were provided by the CESNET LM2015042 and the CERIT Scientific Cloud LM2015085 provided under the program Projects of Large Research, Development, and Innovations Infrastructures. Additional computational resources were obtained from the IT4 Innovations National Supercomputing Center – LM2015070 project supported by MEYS CR from the Large Infrastructures for Research, Experimental Development, and Innovations.

DECLARATION OF INTERESTS

The authors declare no competing interests.

REFERENCES

1. Matsuzaki, K., Y. Mitani, …, K. Miyajima. 1998. Mechanism of synergism between antimicrobial peptides magainin 2 and PGLa. *Biochemistry.* 37:15144–15153.
2. Hara, T., Y. Mitani, …, K. Matsuzaki. 2001. Heterodimer formation between the antimicrobial peptides magainin 2 and PGLa in lipid bilayers: a cross-linking study. *Biochemistry.* 40:12395–12399.
3. Nishida, M., Y. Imura, …, K. Matsuzaki. 2007. Interaction of a magainin-PGLa hybrid peptide with membranes: insight into the mechanism of synergism. *Biochemistry.* 46:14284–14290.
4. Zerweck, J., E. Strandberg, …, A. S. Ulrich. 2017. Molecular mechanism of synergy between the antimicrobial peptides PGLa and magainin 2. *Sci. Rep.* 7:13153.
5. Leber, R., M. Pachler, …, G. Pabst. 2018. Synergism of antimicrobial frog peptides couples to membrane Intrinsic curvature strain. *Biophys. J.* 114:1945–1954.
6. Harmouche, N., and B. Bechinger. 2018. Lipid-mediated interactions between the antimicrobial peptides magainin 2 and PGLa in bilayers. *Biophys. J.* 115:1033–1044.
7. Strandberg, E., J. Zerweck, …, A. S. Ulrich. 2013. Synergistic insertion of antimicrobial magainin-family peptides in membranes depends on the lipid spontaneous curvature. *Biophys. J.* 104:L9–L11.
8. Pachler, M., I. Kabelka, …, G. Pabst. 2019. Magainin 2 and PGLa in bacterial membrane mimics I: peptide-peptide and lipid-peptide interactions. *Biophys. J.* 117:1858–1869.
9. Kabelka, I., M. Pachler, …, R. Vácha. 2020. Magainin 2 and PGLa in bacterial membrane mimics II: membrane fusion and sponge phase formation. *Biophys. J.* 118:612–623.
10. Kabelka, I., V. Georgiev, …, R. Vácha. 2022. Magainin 2 and PGLa in bacterial membrane mimics III: membrane fusion and disruption. *Biophys. J.* 121:852–861.
11. Aisenbrey, C., M. Amaro, …, B. Bechinger. 2020. Highly synergistic antimicrobial activity of magainin 2 and PGLa peptides is rooted in the formation of supramolecular complexes with lipids. *Sci. Rep.* 10:11652.
12. White, S. H., W. C. Wimley, …, K. Hristova. 1998. [4] Protein folding in membranes: determining energetics of peptide-bilayer interactions. *In* Energetics of Biological Macromolecules Part B Elsevier, pp. 62–87.
13. Heerklotz, H., and J. Seelig. 2007. Leakage and lysis of lipid membranes induced by the lipopeptide surfactin. *Eur. Biophys. J.* 36:305–314.
14. Antonny, B. 2011. Mechanisms of membrane curvature sensing. *Ann. Rev. Biochem.* 80:101–123, PMID: 21438688.
15. Campelo, F., and M. M. Kozlov. 2014. Sensing membrane stresses by protein insertions. *PLoS Comput. Biol.* 10:e1003556.
16. Campelo, F., C. Arnarez, …, M. M. Kozlov. 2014. Helfrich model of membrane bending: from Gibbs theory of liquid interfaces to membranes as thick anisotropic elastic layers. *Adv. Coll. Interf. Sci.* 208:25–33.
17. Marx, L., E. F. Semeraro, …, G. Pabst. 2021. Bridging the antimicrobial activity of two lactoferricin derivatives in E. coli and lipid-only membranes. *Front. Med. Technol.* 3:625975.
18. Burstein, E. A., and V. I. Emelyanenko. 1996. Log-normal description of fluorescence spectra of organic fluorophores. *Photochem. Photobiol.* 64:316–320.
19. Ladokhin, A. S., S. Jayasinghe, and S. H. White. 2000. How to measure and analyze tryptophan fluorescence in membranes properly, and why bother? *Anal. Biochem.* 285:235–245.
20. Burstein, E. A., N. S. Vedenkina, and M. N. Ivkova. 1973. Fluorescence and the location of tryptophan residues in protein molecules. *Photochem. Photobiol.* 18:263–279.
21. Wimley, W. C., T. P. Creamer, and S. H. White. 1996. Solvation energies of amino acid side chains and backbone in a family of host-guest pentapeptides. *Biochemistry.* 35:5109–5124.

22. Abraham, M. J., T. Murtola, …, E. Lindahl. 2015. GROMACS: high performance molecular simulations through multi-level parallelism from laptops to supercomputers. *Software.* 1-2:19–25.
23. Páll, S., M. J. Abraham, …, E. Lindahl. 2015. Tackling exascale software challenges in molecular dynamics simulations with GROMACS. *In* Solving Software Challenges for Exascale: International Conference on Exascale Applications and Software, EASC 2014, Stockholm, Sweden, April 2-3, 2014 : Revised Selected Papers. E. Laure and S. Markidis, eds Springer, pp. 3–27, Volume 8759 of LNCS Sublibrary: SL 1 - Theoretical Computer Science and General Issues.
24. Marrink, S. J., H. J. Risselada, …, A. H. de Vries. 2007. The MARTINI force field: coarse grained model for biomolecular simulations. *J. Phys. Chem. B.* 111:7812–7824.
25. Monticelli, L., S. K. Kandasamy, …, S.-J. Marrink. 2008. The MARTINI coarse-grained force field: extension to proteins. *J. Chem. Theor. Comput.* 4:819–834.
26. de Jong, D. H., G. Singh, …, S. J. Marrink. 2013. Improved parameters for the martini coarse-grained protein force field. *J. Chem. Theor. Comput.* 9:687–697.
27. Mandal, T., S. E. Spagnolie, …, Q. Cui. 2021. Protein-induced membrane curvature in coarse-grained simulations. *Biophys. J.* 120:3211–3221.
28. Bussi, G., D. Donadio, and M. Parrinello. 2007. Canonical sampling through velocity rescaling. *J. Chem. Phys.* 126:014101.
29. Parrinello, M., and A. Rahman. 1980. Crystal structure and pair potentials: a molecular-dynamics study. *Phys. Rev. Lett.* 45:1196–1199.
30. Parrinello, M., and A. Rahman. 1981. Polymorphic transitions in single crystals: a new molecular dynamics method. *J. Appl. Phys.* 52:7182–7190.
31. Lan, Y., Y. Ye, …, A. J. Mason. 2010. Structural contributions to the intracellular targeting strategies of antimicrobial peptides. *Biochim. Biophys. Acta Biomembr.* 1798:1934.
32. Bechinger, B., M. Zasloff, and S. Opella. 1998. Structure and dynamics of the antibiotic peptide PGLa in membranes by multidimensional solution and solid-state NMR spectroscopy. *Biophys. J.* 74:981–987.
33. Gesell, J., M. Zasloff, and S. J. Opella. 1997. Two-dimensional 1H NMR experiments show that the 23-residue magainin antibiotic peptide is aαn α-helix in dodecylphosphocholine micelles, sodium dodecylsulfate micelles, and trifluoroethanol/water solution. *J. Biomol. NMR.* 9:127–135.
34. Lee, J., X. Cheng, …, Y. Qi. 2016. CHARMM-GUI input generator for NAMD, GROMACS, AMBER, OpenMM, and CHARMM/OpenMM simulations using the CHARMM36 additive force field. *J. Chem. Theor. Comput.* 12:405–413.
35. Goetz, R., and R. Lipowsky. 1998. Computer simulations of bilayer membranes: self-assembly and interfacial tension. *J. Chem. Phys.* 108:7397–7409.
36. Ollila, O. S., H. J. Risselada, …, S. J. Marrink. 2009. 3D pressure field in lipid membranes and membrane-protein complexes. *Phys. Rev. Lett.* 102:078101.
37. Vanegas, J. M., A. Torres-Sánchez, and M. Arroyo. 2014. Importance of force decomposition for local stress calculations in biomembrane molecular simulations. *J. Chem. Theor. Comput.* 10:691–702.
38. Hu, M., D. H. de Jong, …, M. Deserno. 2013. Gaussian curvature elasticity determined from global shape transformations and local stress distributions: a comparative study using the MARTINI model. *Faraday Discuss.* 161:365–382.
39. Bhaskara, R. M., P. Grumati, …, G. Hummer. 2019. Curvature induction and membrane remodeling by FAM134B reticulon homology domain assist selective ER-phagy. *Nat. Commun.* 10:1–13.
40. Tieleman, D., B. Sejdiu, …, V. Corradi. 2021. Insights into lipid-protein interactions from computer simulations. *Biophys. Rev.* 1–9. https://doi.org/10.1007/s12551-021-00876-9.
41. Gómez-Llobregat, J., F. Elías-Wolff, and M. Lindén. 2016. Anisotropic membrane curvature sensing by amphipathic peptides. *Biophys. J.* 110:197–204.
42. Ben-Shaul, A. 1995. Molecular theory of chain packing, elasticity and lipid-protein interaction in lipid bilayers. *In* Handbook of Biological Physics. R. Lipowsky and E. Sackmann, eds Elsevier, Amterdam, pp. 359–401.
43. Israelachvili, J., S. Marčelja, and R. G. Horn. 1980. Physical principles of membrane organization. *Q. Rev. Biophys.* 13:121–200.
44. Sodt, A. J., and R. W. Pastor. 2014. Molecular modeling of lipid membrane curvature induction by a peptide: more than simply shape. *Biophys. J.* 106:1958–1969.
45. Venable, R. M., F. L. H. Brown, and R. W. Pastor. 2015. Mechanical properties of lipid bilayers from molecular dynamics simulation. *Chem. Phys. Lipids.* 192:60–74.
46. Pabst, G., S. Danner, …, J. Katsaras. 2007. Entropy-driven softening of fluid lipid bilayers by alamethicin. *Langmuir.* 23:11705–11711.
47. Chang, W.-F., S.-H. Chen, and Y.-F. Chen. 2018. Correlation of an antimicrobial peptide's potency and its influences on membrane elasticity. *Phys. Rev. E.* 98:042408.
48. Seelig, J. 2004. Thermodynamics of lipid-peptide interactions. *Biochim. Biophys. Acta.* 1666:40–50.
49. Koller, D., and K. Lohner. 2014. The role of spontaneous lipid curvature in the interaction of interfacially active peptides with membranes. *Biochim. Biophys. Acta Biomembr.* 1838:2250–2259, Interfacially Active Peptides and Proteins.
50. Wessman, P., M. Morin, …, K. Edwards. 2010. Effect of -helical peptides on liposome structure: a comparative study of melittin and alamethicin. *J Colloid Interf Sci.* 346:127–135.
51. Larsen, J. B., K. R. Rosholm, …, D. Stamou. 2020. How membrane geometry regulates protein sorting independently of mean curvature. *ACS Cent. Sci.* 6:1159–1168. https://doi.org/10.1021/acscentsci.0c00419.

Biophysical Journal, Volume *121*

Supplemental information

Magainin 2 and PGLa in bacterial membrane mimics IV: Membrane curvature and partitioning

Enrico F. Semeraro, Peter Pajtinka, Lisa Marx, Ivo Kabelka, Regina Leber, Karl Lohner, Robert Vácha, and Georg Pabst

SUPPLEMENTARY MATERIAL

Magainin 2 and PGLa in Bacterial Membrane Mimics IV: Membrane Curvature and Partitioning

E.F. Semeraro, P. Patik, L. Mark, I. Kableka, R. Leber, K. Lohner, R. Vácha, and G. Pabst

Table S1: List of simulated egg-box bilayers. The overall simulation length, number of peptide copies per leaflet, and height amplitude, Amp, is listed.

Peptide	Amp [nm]	#Replicas	Length [μs]	#Peptides per leaflet
MG2a	1.0	1	10.5	2
MG2a	1.0	2	6.75	2
MG2a	2.5	1	10.5	2
MG2a	2.5	2	4.5	2
MG2a	4.0	1	10.5	2
MG2a	4.0	2	4.5	2
L18W-PGLa	1.0	1	10.5	2
L18W-PGLa	1.0	2	6.75	2
L18W-PGLa	2.5	1	10.5	2
L18W-PGLa	2.5	2	4.5	2
L18W-PGLa	4.0	1	10.5	2
L18W-PGLa	4.0	2	4.5	2
MG2a:MG2a	2.5	1	10.5	4 (2 dimers)
L18W-PGLa:L18W-PGLa	2.5	1	10.5	4 (2 dimers)
L18W-PGLa:MG2a	2.5	1	10.5	4 (2 dimers)
L18W-PGLa:MG2a	2.5	2	5.25	4 (2 dimers)

Table S2: Peptide-induced changes of mean torque, $\Delta\tau$, and spontaneous monolayer curvature, Δc_0^m for peptides with imposed fully or partial α-helical secondary structure. Reported values are averaged over both symmetric lipid leaflets.

Peptides	$\Delta\tau$ [10^{-12} J m^{-1}]	Δc_0^m [nm^{-1}]
MG2a[a,c]	1.88 ± 0.15	0.029 ± 0.003
MG2a[a,d]	1.7 ± 0.3	0.027 ± 0.004
PGLa[a,c]	0.98 ± 0.18	0.015 ± 0.003
PGLa[a,e]	0.92 ± 0.11	0.014 ± 0.002
2x MG2a[b,c]	3.7 ± 0.6	0.057 ± 0.009
2x MG2a[b,d]	3.1 ± 0.5	0.049 ± 0.007
2x PGLa[b,c]	2.3 ± 0.9	0.036 ± 0.014
2x PGLa[b,e]	2.58 ± 0.16	0.040 ± 0.003
L18W-PGLa:MG2a[b,c,c]	2.75 ± 0.19	0.043 ± 0.004
L18W-PGLa:MG2a[b,e,d]	2.77 ± 0.14	0.043 ± 0.003

[a] monomer.
[b] dimer
[c] 100% helicity.
[d] 74% helicity.
[e] 76% helicity.

Table S3: Average value of the sensed mean membrane curvature, H, by studied peptides with different helical content. Standard errors were calculated from sampled curvatures by peptides on different leaflets and different systems.

Peptides	H [nm^{-1}]
MG2a (monomer)[a]	0.0848 ± 0.0006
MG2a (monomer)[b]	0.0821 ± 0.0013
PGLa (monomer)[a]	0.065 ± 0.002
PGLa (monomer)[c]	0.075 ± 0.002
2x MG2a (dimer)[a,d]	0.1033 ± 0.0003
2x MG2a (dimer)[b]	0.1003 ± 0.0007
2x PGLa (dimer)[a]	0.0859 ± 0.0004
2x PGLa (dimer)[c]	0.0933 ± 0.0010
L18W-PGLa:MG2a (dimer)[a,d]	0.1017 ± 0.0003
L18W-PGLa:MG2a (dimer) [c,b,d]	0.0990 ± 0.0006

[a] 100% helicity.
[b] 74% helicity.
[c] 76% helicity.
[d] One of the dimers dissociated during the simulation run and was therefore discarded from the analysis.

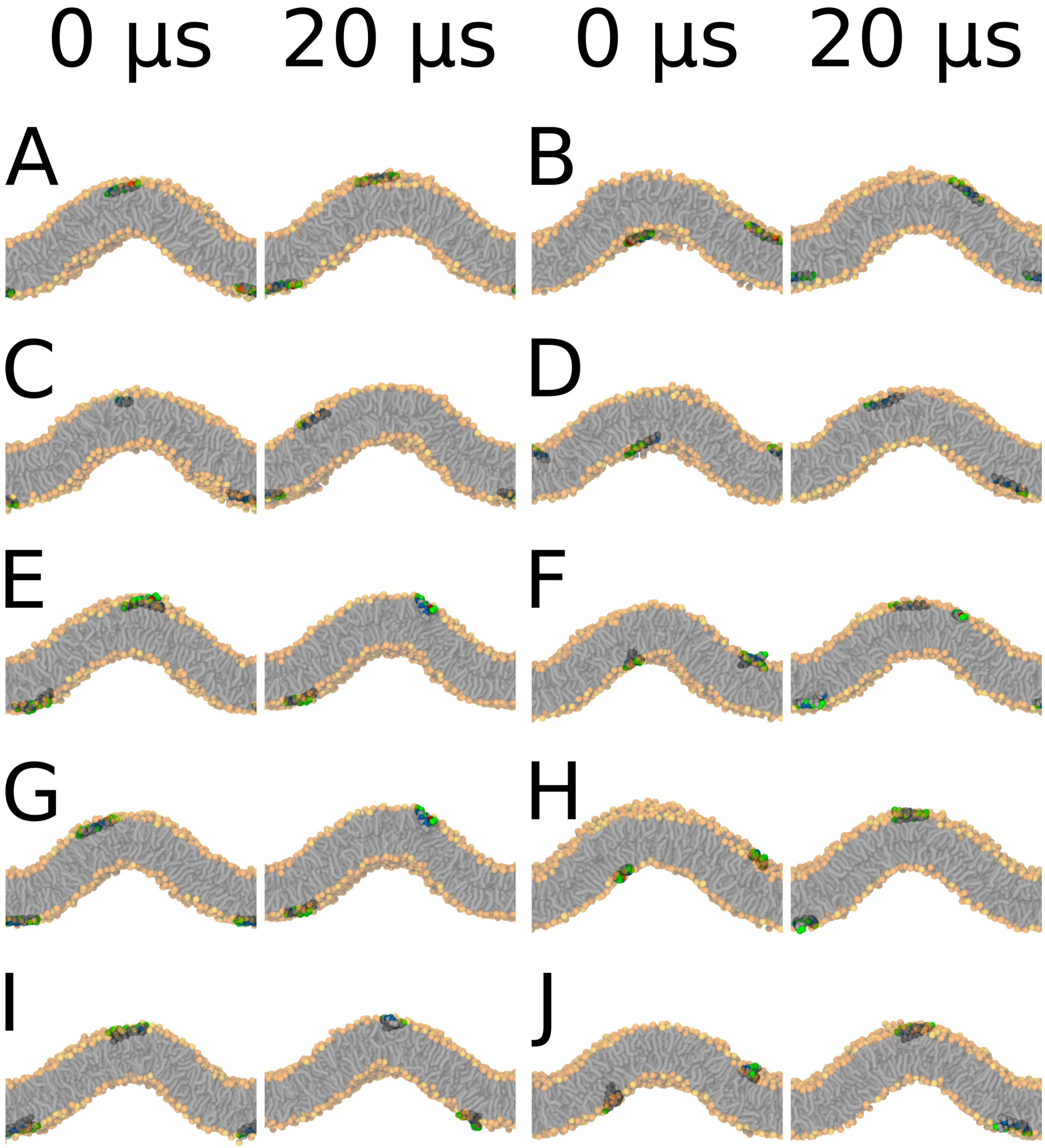

Figure S1: Initial and final simulation snapshots of peptide localization on a curved PE:PG (3:1 mol/mol) membrane. (*A*–*B*) two different initial configurations of systems comprising monomeric MG2a on each membrane leaflet (*C*–*D*) two different initial configurations of monomeric L18W-PGLa, one on each membrane leaflet. (*E*–*F*) two different initial configurations of systems with heterodimer MG2a:L18W-PGLa, one heterodimer on each leaflet (*G*–*H*) two different initial configurations of systems with MG2a homodimer, one homodimer on each leaflet (*I*–*J*) two different initial configurations of systems with L18W-PGLa homodimer, one homodimer on each leaflet.

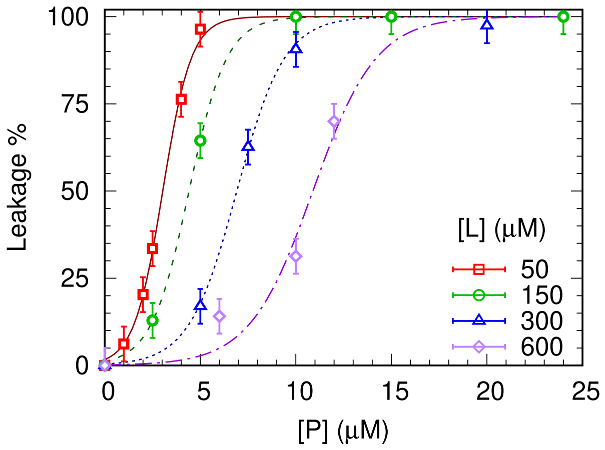

Figure S2: Leakage percentage of the dye-quencher pair ANTS/DPX from POPE/POPG 3:1 lipid vesicles as a function L18W-PGLa concentration, $[P]$, at different total lipid concentrations $[L]$. Data were fitted with a sigmoidal function by fixing a final plateau at 100%.

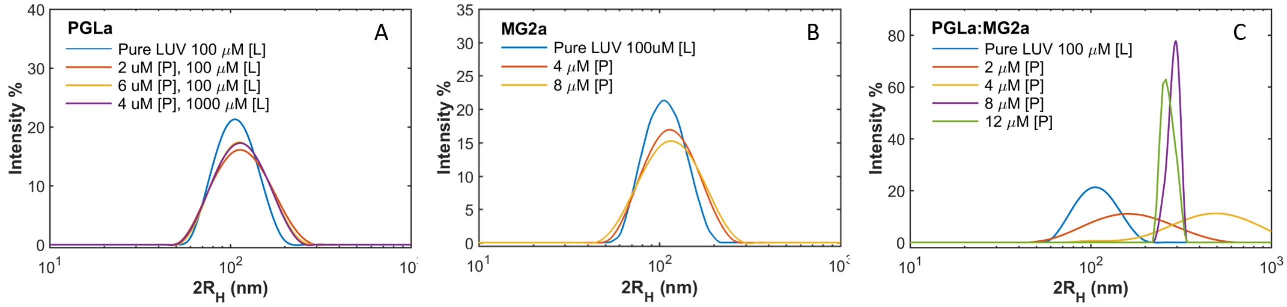

Figure S3: Representative size distribution functions (intensity-weighted) of POPE/POPG 3:1 membrane mimics after incubation with peptides for one hour at 37 °C. **A:** LUVs ($[L]$=100 or 1000 μM) after incubation with PGLa ($[P]$=2-6 μM). **B:** ($[P]$=4-8 μM) MG2a mixed with LUVs ($[L]$=100 μM). **C:** ($[P]$=2-12 μM) PGLa:MG2a mixed with LUVs ($[L]$=100 μM).

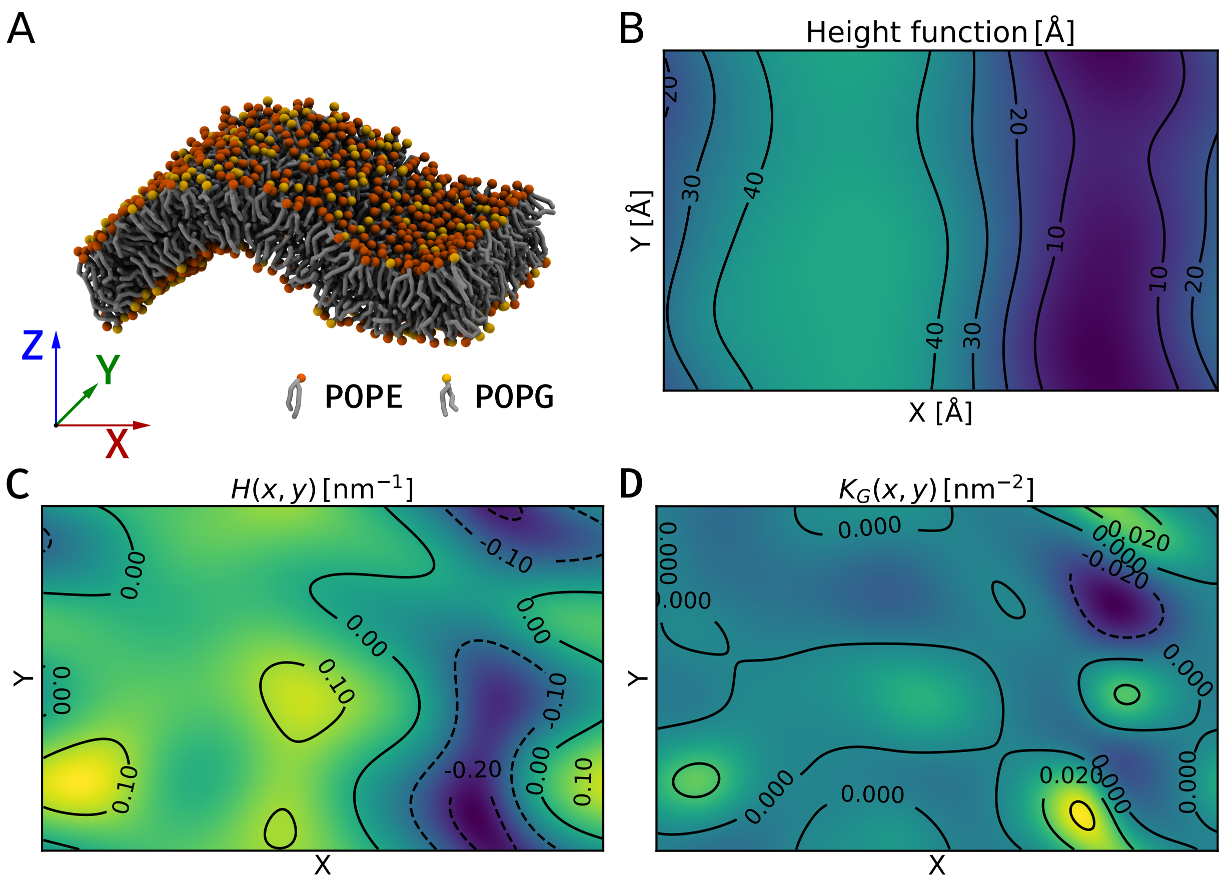

Figure S4: A snapshot of buckled bilayer system (*A*) with a corresponding map of height function (*B*), map of mean curvature (*C*), and a map of Gaussian curvature (*D*). In the snapshot, solvent, ion, and protein beads are omitted for clarity. The maps shown here correspond to the upper membrane leaflet.

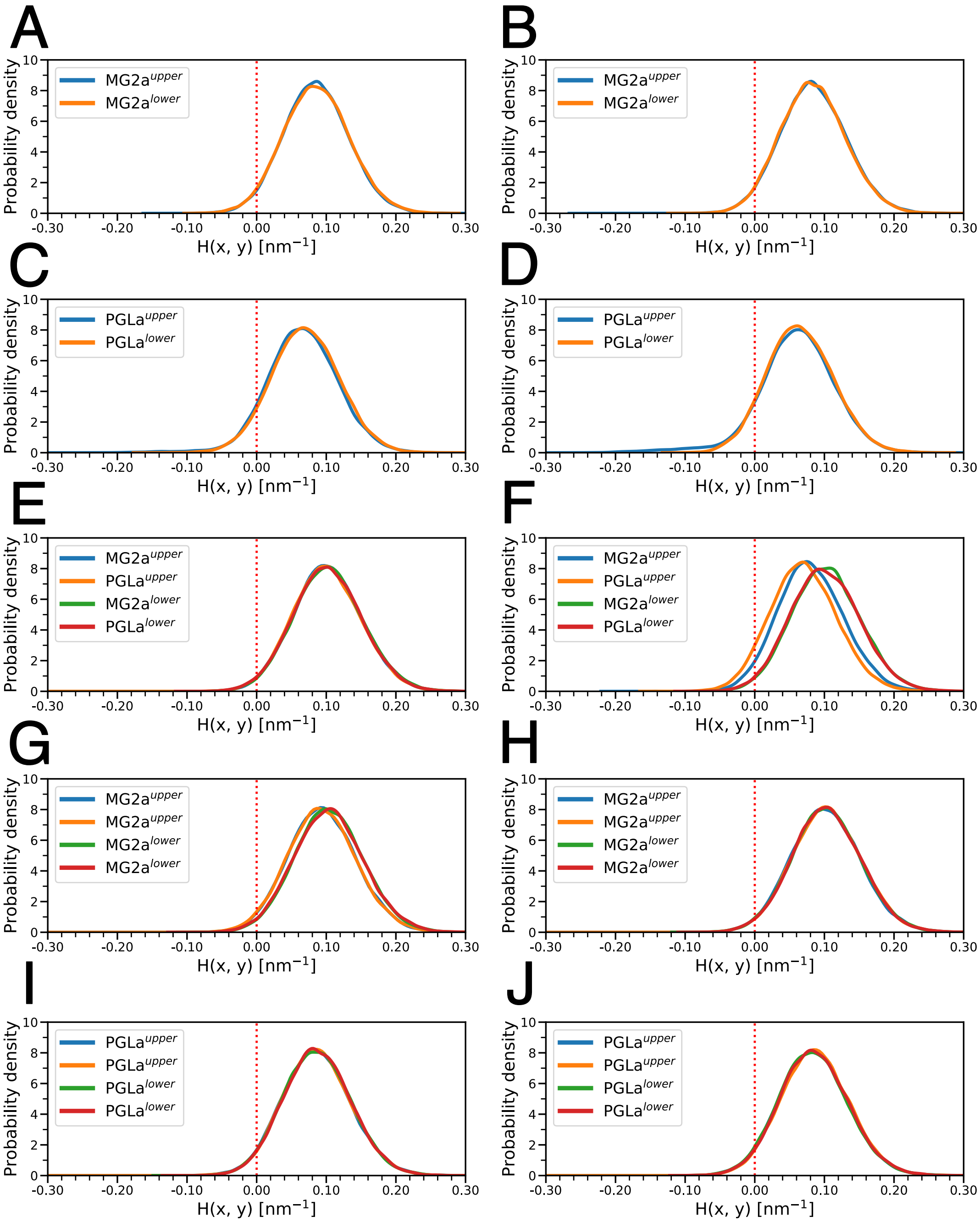

Figure S5: Kernel density estimates from the histograms of the sampled mean curvature by peptides, with fully α-helical secondary structure, on the buckled PE:PG (3:1 mol/mol) membrane. The red dotted-line highlights the zero mean curvature. (*A*–*B*) two different initial configurations of systems comprising monomeric MG2a on each membrane leaflet. (*C*–*D*) two different initial configurations of monomeric L18W-PGLa, one on each membrane leaflet. (*E*–*F*) two different initial configurations of systems with heterodimer MG2a:L18W-PGLa, one heterodimer on each leaflet. In the second system, one copy of heterodimer disassembled (after approximately 4 μs), resulting in a different curvature sampled by the MG2a and L18W-PGLa monomers. (*G*–*H*)-two different initial configurations of homodimer of MG2a. Similarly as in the case of heterodimer, one copy of dimer dissociated (after approx. 12 μs) and different mean curvatures sampled by the monomers caused slight shift in the distribution towards lower values of mean curvature. (*I*–*J*)-two initial configurations of PGLa homodimer.

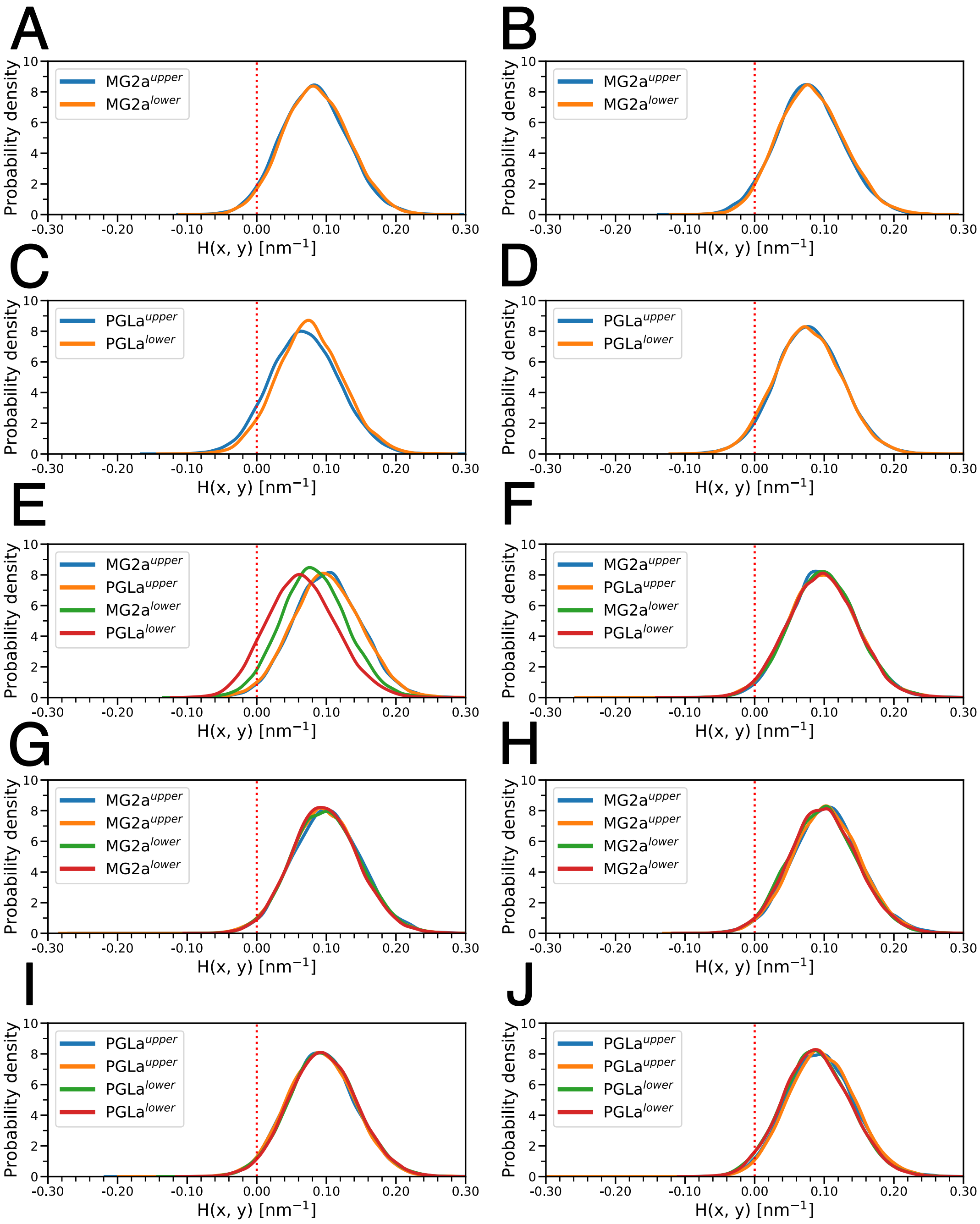

Figure S6: Kernel density estimates from the histograms of the sampled mean curvature by peptides, with partially unfolded secondary structure (for details see Methods section), on the buckled PE:PG (3:1 mol/mol) membrane. The red dotted-line highlights the zero mean curvature. (*A–B*) two different initial configurations of systems comprising monomeric MG2a on each membrane leaflet. (*C–D*) two different initial configurations of monomeric L18W-PGLa, one on each membrane leaflet. (*E–F*) two different initial configurations of systems with heterodimer MG2a:L18W-PGLa, one heterodimer on each leaflet. In the first system, one copy of heterodimer disassembled within the first microsecond, resulting in a different curvature sampled by the MG2a and L18W-PGLa monomers. (*G–H*)-two different initial configurations of homodimer of MG2a. (*I–J*)-two initial configurations of PGLa homodimer.

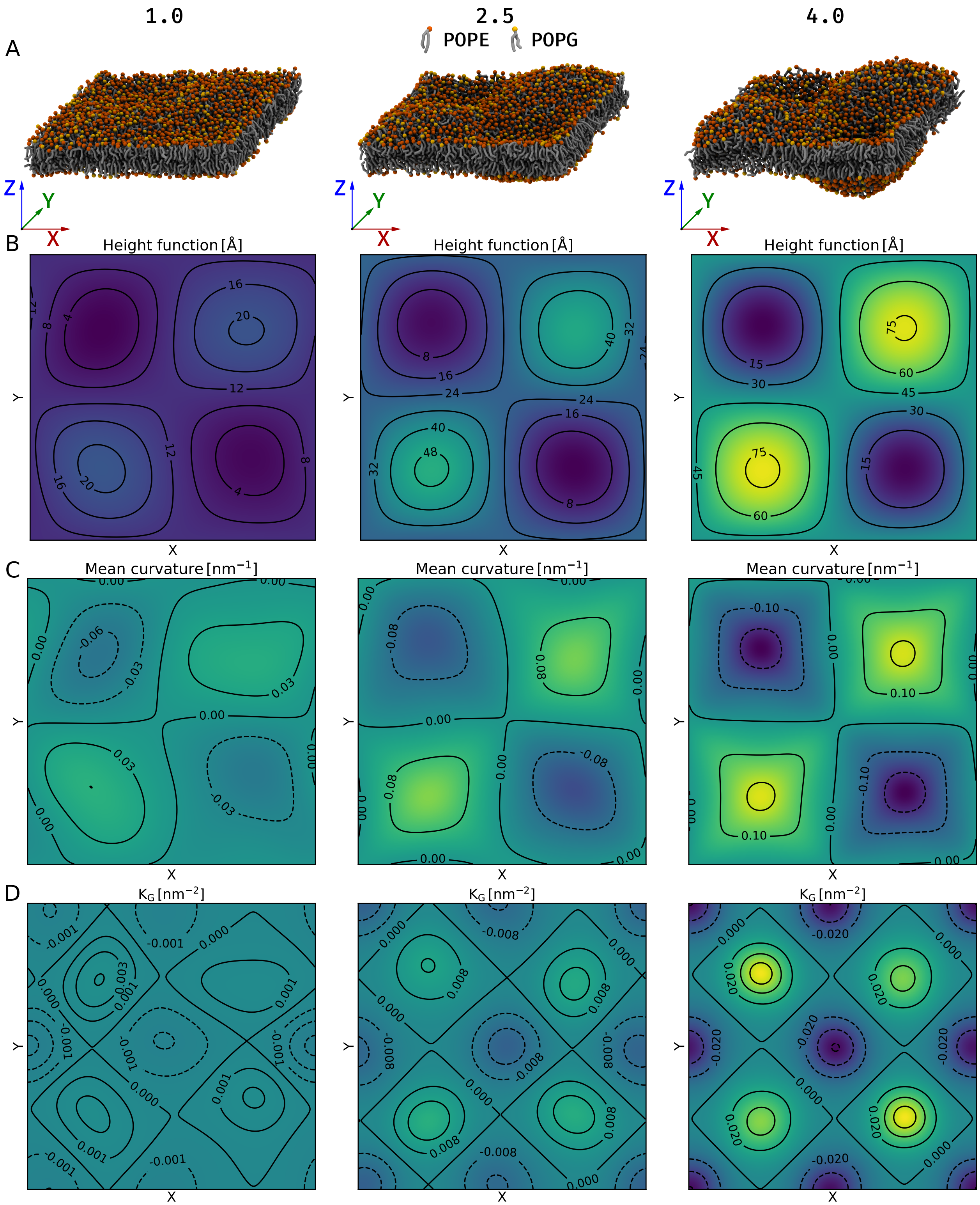

Figure S7: An illustrative summary of the egg-box bilayer systems with different height amplitudes (1.0, 2.5, and 4.0 nm) and corresponding accessible curvatures. In the top row (*A*), snapshots for systems of different amplitudes are shown. The corresponding maps of height function (*B*), mean curvature distribution (*C*), and Gaussian curvature distribution (*D*) for each of the amplitudes are shown in the appropriate column.

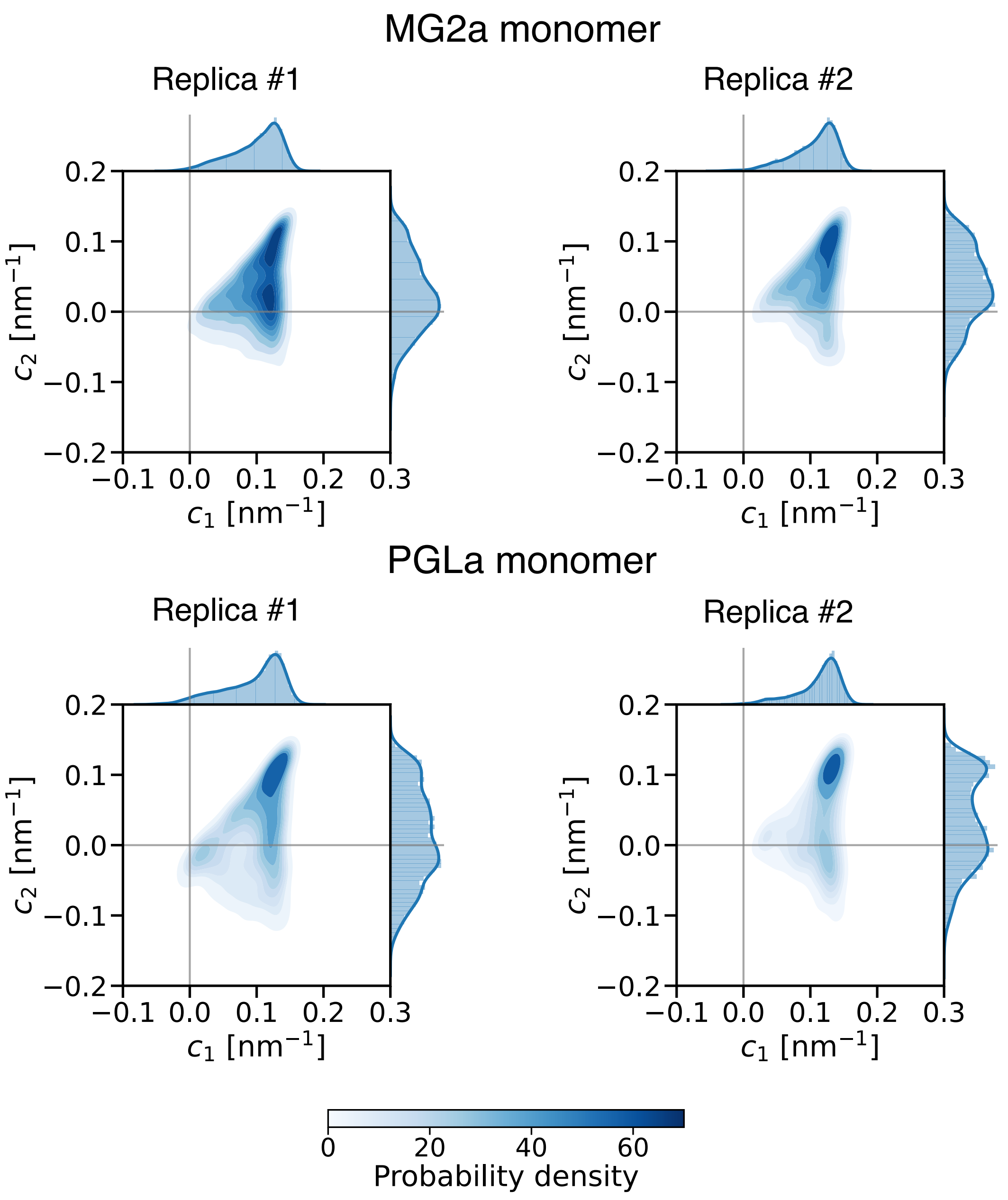

Figure S8: 2D histograms of principal curvatures c_1 and c_2 sampled by the monomeric MG2a and PGLa peptides on the egg-box shaped bilayer. Results obtained from both replicas are presented side by side. Gaussian curvature, K_G, is defined as c_1c_2.

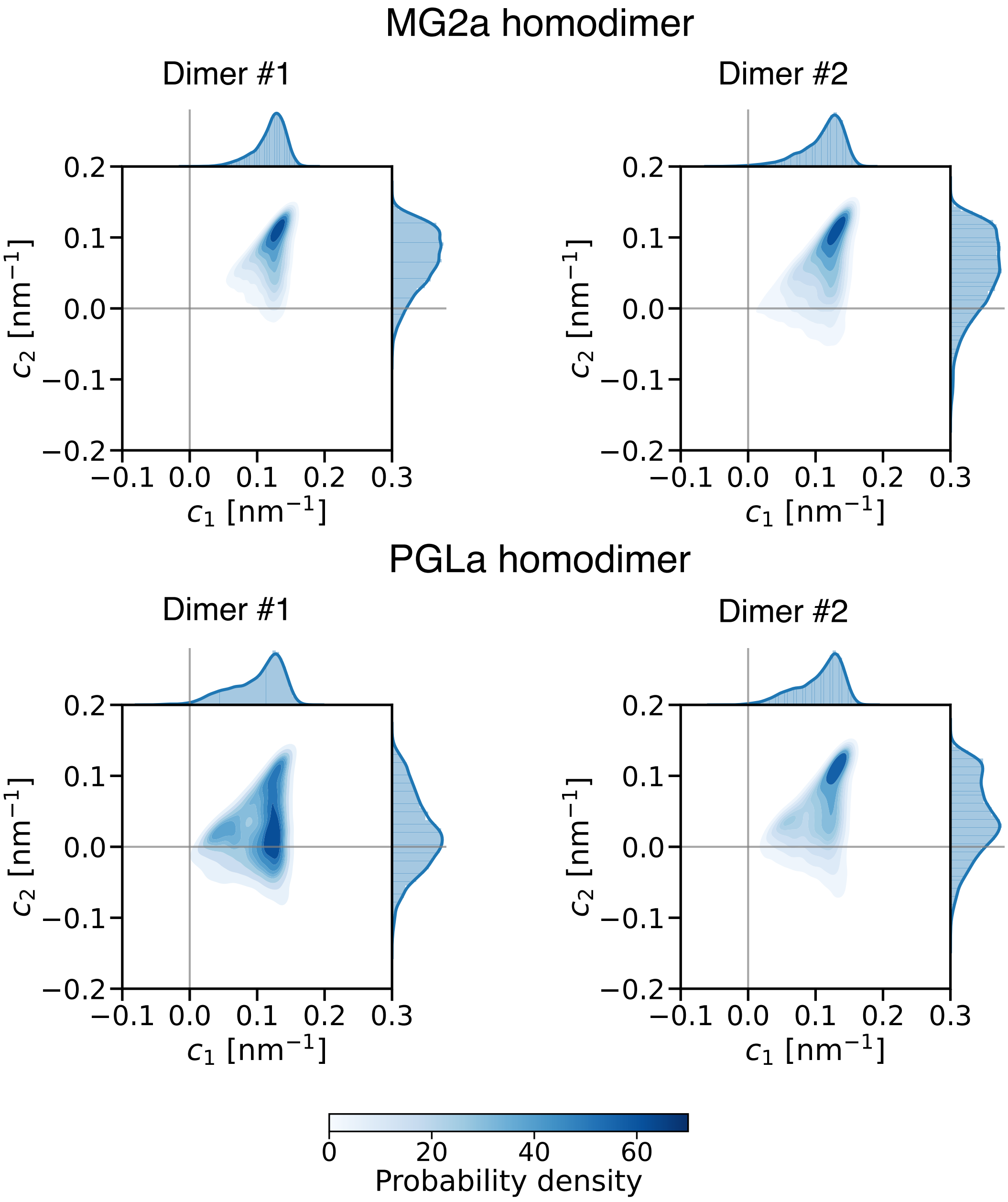

Figure S9: 2D histograms of principal curvatures c_1 and c_2 sampled on the egg-box shaped bilayer by the homodimeric MG2a and PGLa peptides, respectively. Results obtained from both dimers present on unbiased leaflet are shown side by side. Gaussian curvature, K_G, is defined as $c_1 c_2$.

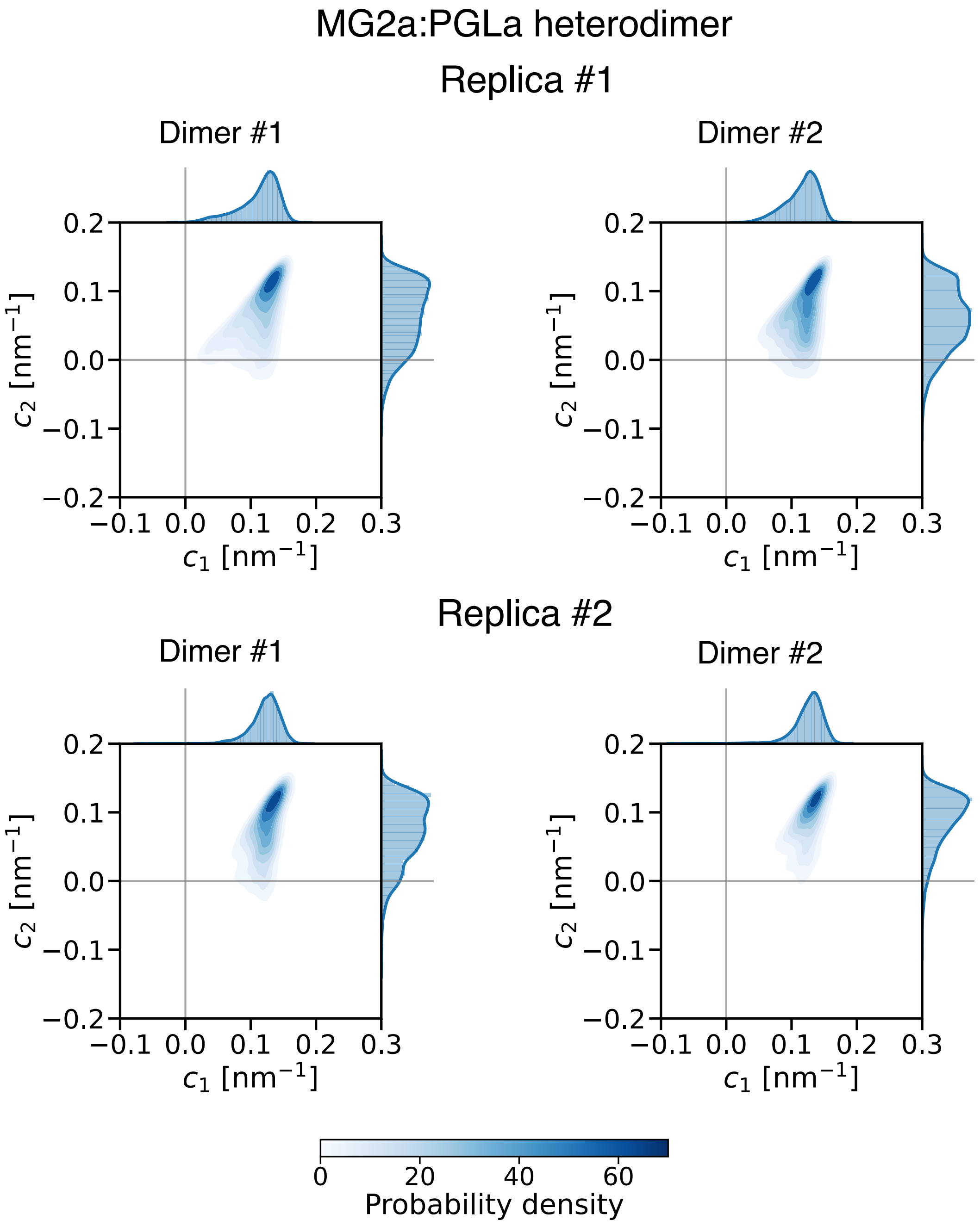

Figure S10: 2D histograms of principal curvatures c_1 and c_2 sampled on the egg-box shaped bilayer by the MG2a:PGLa heterodimer. Results obtained from both replicas and from both dimers present at unbiased membrane leaflets are shown. Gaussian curvature, K_G, is defined as c_1c_2.

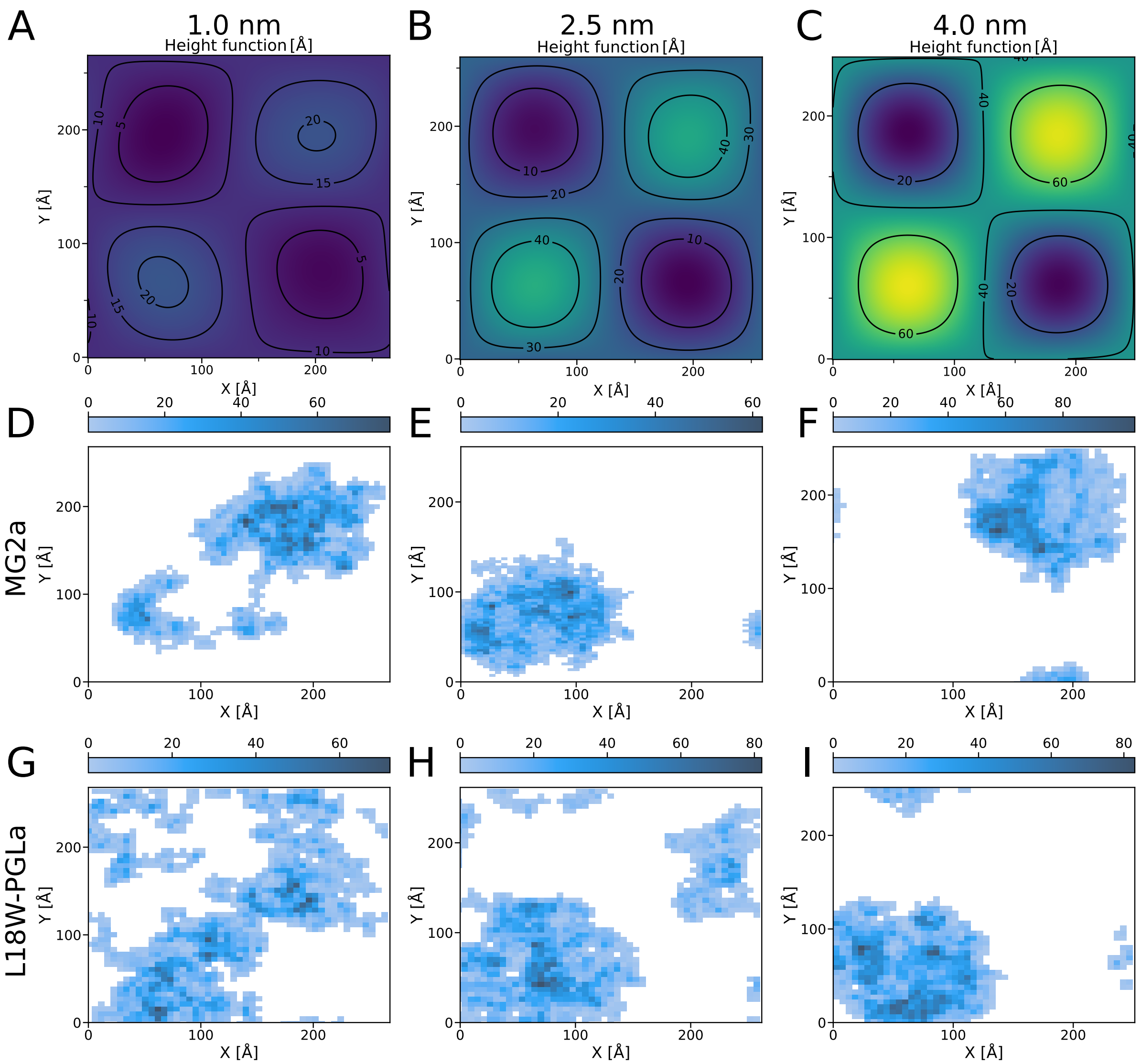

Figure S11: Comparison of the systems with egg-box shaped membranes of different amplitudes (1.0 nm, 2.5 nm, or 4.0 nm). To investigate the effect, systems containing one copy of monomeric MG2a or L18W-PGLa were used. (*A*-*C*) Height functions, representing the shape of the upper leaflet for different amplitudes are shown. 2D histograms of XY coordinates MG2a center of mass sampled during the simulation run at membrane with amplitude 1.0 nm (*D*), 2.5 nm (*E*), and 4.0 nm (*F*). 2D histogram of XY coordinates L18W-PGLa center of mass visited during the simulation run on membrane with 1.0 nm (*G*), 2.5 nm (*H*), and 4.0 nm (*I*) amplitude.

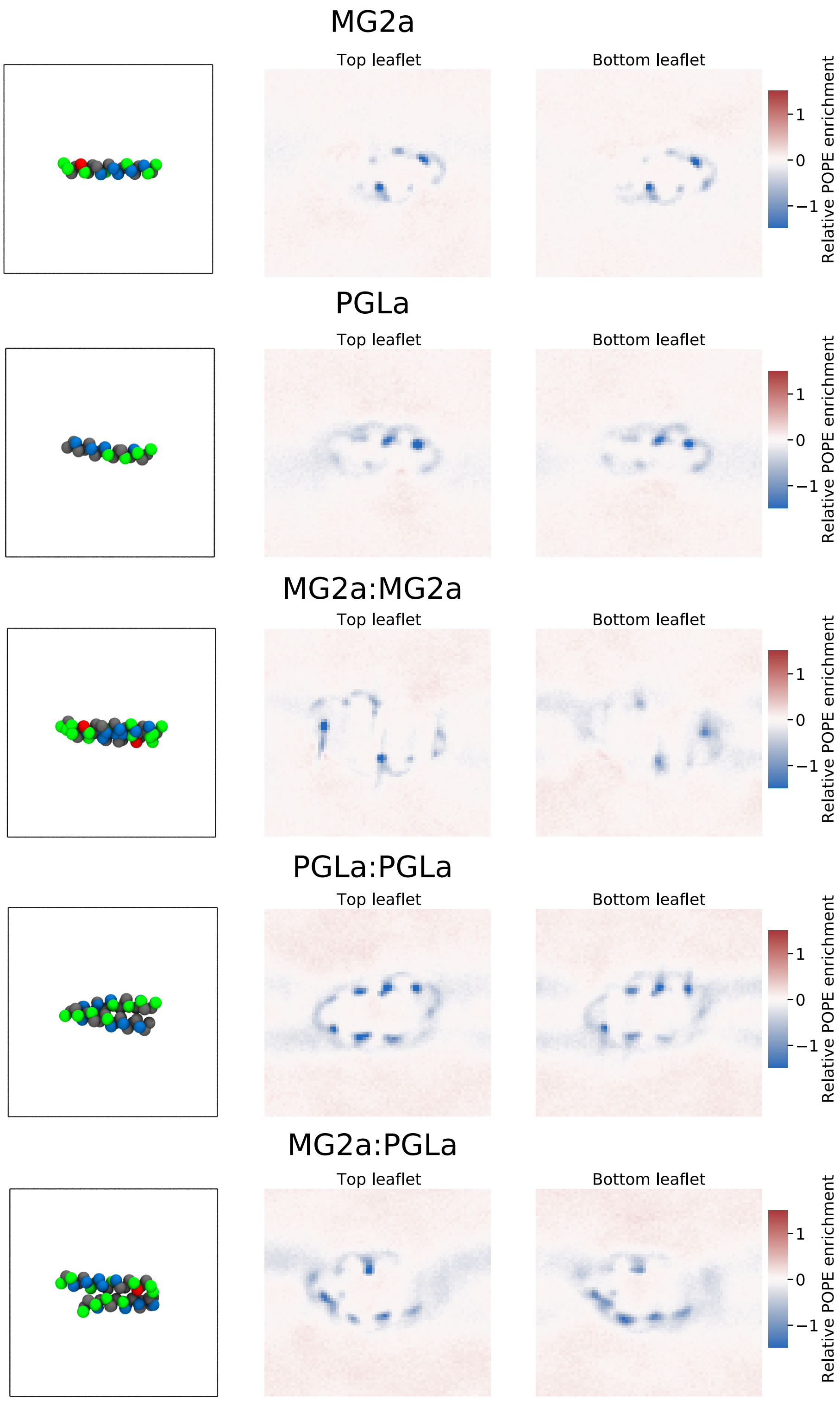

Figure S12: 2D averaged density maps of a planar POPE:POPG (3:1 mol:mol) membrane with peptides averaged over an 8μs trajectory, showing relative POPE enrichment in the proximity of the peptides. Negative POPE enrichment corresponds to the enrichment of POPG in the particular region. The systems were centered and aligned on peptides as indicated by the snapshots. The enrichment was calculated as a difference between normalized density maps of PO_4 beads from POPE and POPG lipids. Data were averaged over both membrane leaflets.

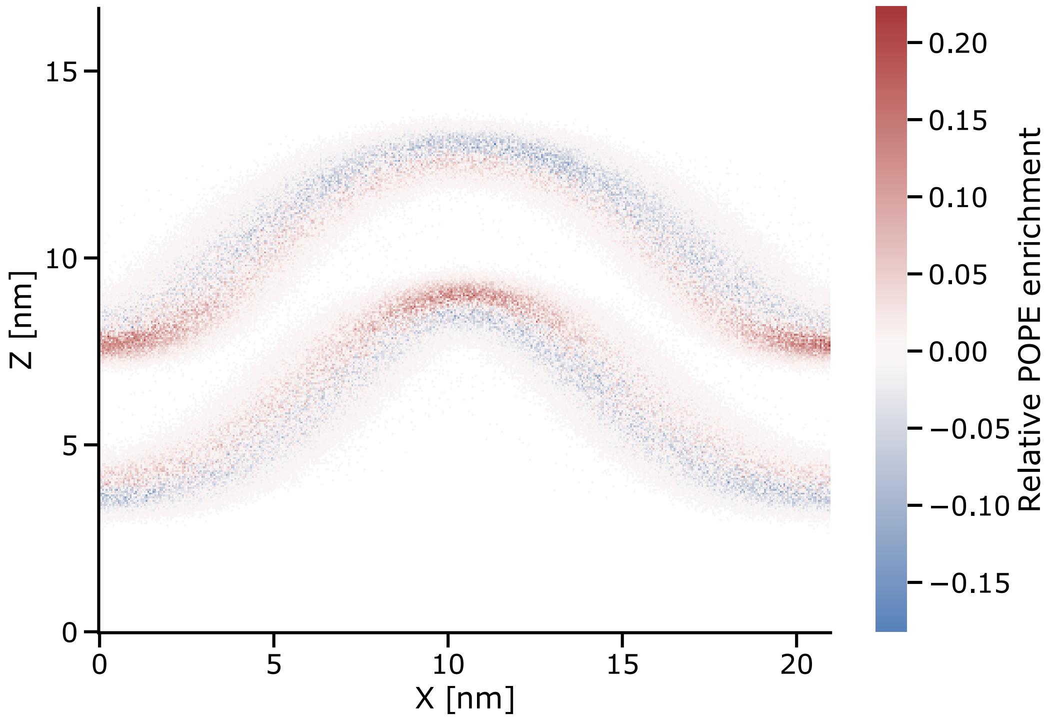

Figure S13: Density map of a membrane POPE:POPG (3:1 mol:mol) buckle without peptides averaged over Y direction, showing relative POPE enrichment within differently curved membrane regions. The enrichment was calculated as a difference between normalized density profiles of POPE and POPG lipids. Note that only densities of PO4 beads are shown.

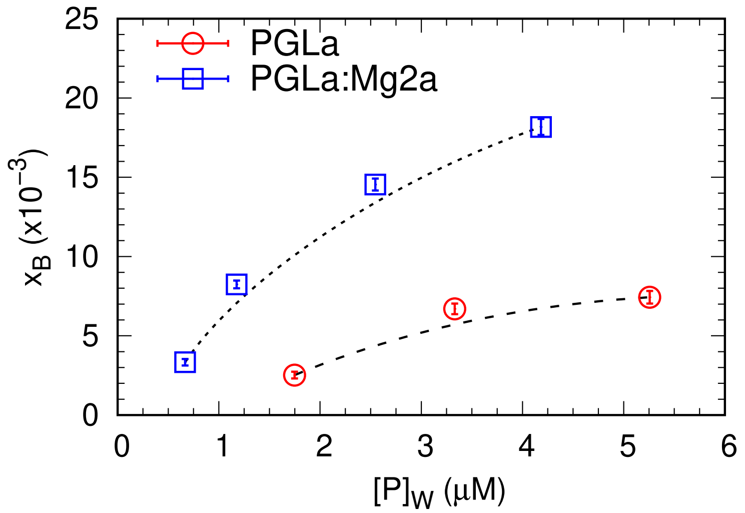

Figure S14: Number of partitioned L18W-PGLa per lipid molecule, x_B, as a function of free PGLa in bulk, $[P]_W$, for $[L] = 100$ μM at different PGLa concentrations. Dashed lines are guides for the eyes.

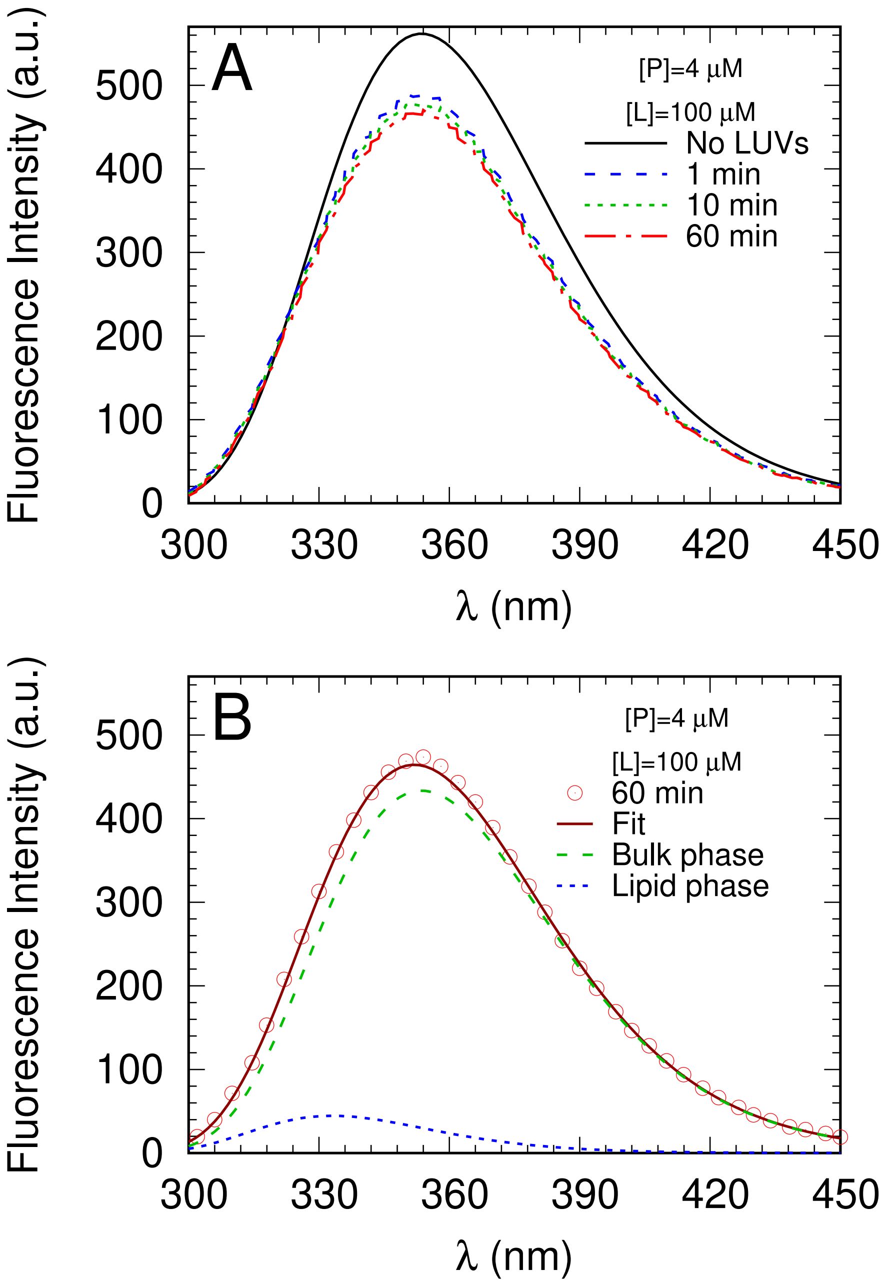

Figure S15: **A:** kinetics of the Trp emission band of L18W-PGLa upon partitioning into the lipid bilayer. The plot shows an example at $[P] = 4\ \mu$M and POPE/POPG 3:1 total lipid concentration $[L] = 100\ \mu$M. **B:** best fit (solid red line) of the spectrum after 60 minutes of incubation at $[P] = 4\ \mu$M and $[L] = 100\ \mu$M [see (17) for details]. The curve is well described by the sum of the emission band from PGLa in the aqueous phase (dashed green line) and in the lipid phase (dotted blue line).

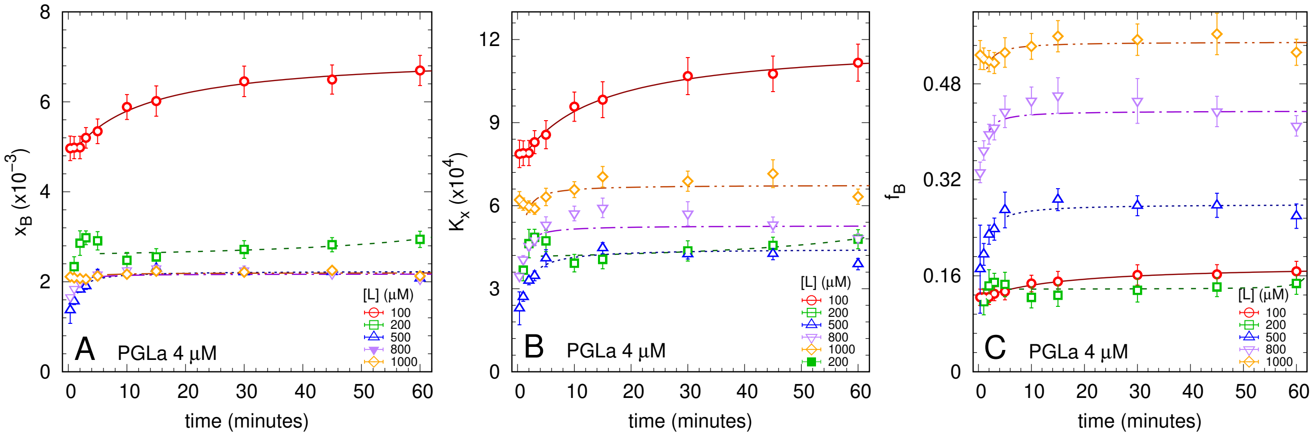

Figure S16: Kinetics of **A:** R_B, **B:** K and **C:** f_B at different POPE/POPG 3:1 concentrations and fixed L18W-PGLa $[P] = 4$ μM. The lines are just guides for the eyes. Every combination reaches a plateau within < 10 minutes, with the exception of the lower $[L]$ tested (100 μM), where partitioning parameters slowly plateau in the course of about 1 hour.
